# Herbal Medicine for Traumatic Brain Injury: A Systematic Review and Meta-Analysis of Randomized Controlled Trials and Limitations

**DOI:** 10.3389/fneur.2020.00772

**Published:** 2020-09-18

**Authors:** Boram Lee, Jungtae Leem, Hyunho Kim, Hee-Geun Jo, Chan-Young Kwon

**Affiliations:** ^1^Clinical Medicine Division, Korea Institute of Oriental Medicine, Daejeon, South Korea; ^2^Research and Development Institute, CY Pharma Co., Seoul, South Korea; ^3^Chung-Yeon Central Institute, Gwangju, South Korea; ^4^Chung-Yeon Korean Medicine Hospital, Gwangju, South Korea; ^5^Department of Oriental Neuropsychiatry, Dong-eui University College of Korean Medicine, Busan, South Korea

**Keywords:** herbal medicine, traumatic brain injuries, systematic review, East Asian traditional medicine, post-concussion syndrome

## Abstract

**Background:** This systematic review aimed to evaluate the effectiveness (functional outcomes and clinical symptoms) and safety (incidence of adverse events) of herbal medicine (HM) as monotherapy or adjunctive therapy to conventional treatment (CT) for traumatic brain injury (TBI).

**Methods:** We comprehensively searched 14 databases from their inception until July 2019. Randomized controlled trials (RCTs) using HM as monotherapy or adjunctive therapy to treat TBI patients were included. The primary outcome was functional outcomes, consciousness state, morbidity, and mortality. Meta-analysis was performed to calculate a risk ratio (RR) or mean difference (MD) with 95% confidence intervals (CIs), when appropriate data were available. Methodological quality of RCTs and the strength of evidence were also assessed.

**Results:** Thirty-seven RCTs with 3,374 participants were included. According to meta-analysis, HM as a monotherapy (RR 1.29, 95% CI: 1.21–1.37) or an adjunctive therapy to CT (RR 1.21, 95% CI: 1.16–1.27) showed significantly better total effective rate based on clinical symptoms, compared to CT alone. Subgroup analysis showed that HM had significantly improved post-concussion syndrome, dizziness, headache, epilepsy, and mild TBI, but not traumatic brain edema, compared to CT. Moreover, HM combined with CT had significantly improved post-concussion syndrome, mental disorder, headache, epilepsy, and mild TBI-like symptoms, but not cognitive dysfunction and posttraumatic hydrocephalus, compared to CT alone. When HM was combined with CT, functional outcomes such as activities of daily living and neurological function were significantly better than in patients treated using CT alone. In terms of the incidence of adverse events, HM did not differ from either CT (RR 0.88, 95% CI: 0.33–2.30) or placebo (RR 2.29, 95% CI: 0.83–6.32). However, HM combined with CT showed better safety profile than CT alone (RR 0.64, 95% CI: 0.44–0.93). Most studies had a high risk of performance bias, and the quality of evidence was mostly rated “very low” to “moderate,” mostly because the included studies had a high risk of bias and imprecise quantitative synthesis results.

**Conclusion:** The current evidence suggests that there is insufficient evidence for recommending HM for TBI in clinical practice. Therefore, further larger, high-quality, rigorous RCTs should be conducted.

## Introduction

External force to the head can cause varying degrees of organic and/or functional abnormalities in the brain, ranging from mild to fatal. Traumatic brain injury (TBI) can be defined as “an alteration in brain function, or other evidence of brain pathology, caused by an external force” (1). TBI is a major threat to public health worldwide. In particular, this condition is an important cause of death and hospitalization (2). According to data from the Centers for Disease Control and Prevention (CDC) (3), the most common external causes of TBI are falls (common in childhood and in the elderly) and road traffic accidents (common in young adults). These results were confirmed in epidemiological studies carried out in Europe (2, 4). A recent systematic review of 82 population-based studies reporting the worldwide prevalence of TBI concluded that approximately 300 cases per 100,000 people occur per year, especially in Asia, with about 380 cases per 100,000, which is higher than the worldwide average (5).

Depending on the area and severity of the initial trauma, the severity of TBI can vary and is classified as mild, moderate, or severe using tools like the Glasgow Coma Scale (GCS) (6), which is based on the patient's state of consciousness (6). Many patients with TBI, even mild TBI, experience post-concussion syndrome (PCS), which involves a complex of symptoms including headache, dizziness, cognitive impairment, and neuropsychiatric symptoms (7). Moreover, TBI can cause persistent, sometimes life-long consequences, even in moderate or mild cases, and it can be associated with long-term negative outcomes that markedly reduce quality of life (QoL) of survivors, such as excess mortality, vegetative state, physical disability, cognitive impairment, depression, anxiety, psychosis, and seizures (8). In addition, TBI may be related to neurodegenerative diseases such as dementia (9), but not Parkinson's disease (10).

According to the CDC report (3), nearly half of patients with moderate-to-severe TBI undergoing inpatient rehabilitation experience pathological changes in their cognitive function between 1 and 5 years after injury (11). Therefore, to prevent long-term negative consequences and improve QoL, TBI requires long-term management as well as acute, post-injury treatment.

Complementary and integrative medicine (CIM) approaches, including acupuncture and herbal medicine (HM), are often used to supplement the limitations of conventional medicine (12, 13), improve effectiveness, and sometimes reduce side effects, even in the management of TBI (14, 15). In particular, HM has been used to manage brain trauma such as hemorrhage-related hydrocephalus (16), as well as long-term neurological diseases such as stroke (17), cerebral palsy (18), Parkinson's disease (19), vascular dementia (20), and Alzheimer's disease (21). In the field of brain trauma, common HMs such as *Goreisan* have been shown to prevent chronic subdural hematoma recurrence (22, 23), and the mechanism may involve the regulation of aquaporin, a water channel (24–26). Similarly, some HMs such as *Yokukansan* (27) and *Xuefu Zhuyu* decoction (28) have beneficial effects on TBI-related behavioral changes or cognitive impairment. In the management of TBI, HMs may have beneficial effects through complex mechanisms; they may reduce tumor necrosis factor-α or nitric oxide expression, improve blood-brain-barrier permeability, and reduce brain water content (29). However, no studies have yet synthesized all the clinical evidence for the effectiveness and safety of HM as an adjunctive or alternative therapy for various outcomes of TBI, including functional outcomes (mobility and global disability), mortality, quality of life, global clinical improvement, and adverse events. The present systematic review aimed to evaluate the effectiveness and safety of HM on these outcomes in TBI compared to placebo, no treatment, and conventional treatment (CT), to inform clinicians, policy makers, and patients in how to manage this disease.

## Methods

### Study Registration

The protocol of this systematic review has been published and registered in PROSPERO (registration number, CRD42018116559) (30), and the study was reported in accordance with the Preferred Reporting Items for Systematic Reviews and Meta-Analyses (PRISMA) statement (31) and the Cochrane Handbook for Systematic Reviews of Interventions (32).

### Data Sources and Search Strategy

As previously described, the following 14 databases were searched comprehensively: five English-language databases (Medline via PubMed, EMBASE via Elsevier, the Cochrane Central Register of Controlled Trials [CENTRAL], the Allied and Complementary Medicine Database [AMED] via EBSCO, and the Cumulative Index to Nursing and Allied Health Literature [CINAHL] via EBSCO), five Korean-language databases (Oriental Medicine Advanced Searching Integrated System [OASIS], Korean studies Information Service System [KISS], Research Information Service System [RISS], Korean Medical Database [KMbase], and Korea Citation Index [KCI]), three Chinese-language databases (China National Knowledge Infrastructure [CNKI], Wanfang Data, and VIP), and one Japanese database (CiNii). The initial search date was December 2, 2018 and we conducted an updated search on July 27, 2019 to retrieve more up-to-date and comprehensive evidence. Additionally, we searched the reference lists of the relevant articles and performed a manual search on Google Scholar to identify further eligible studies. We also included “gray literature,” such as degree theses and conference proceedings, as well as the literature published in journals. There was no restriction on language, publication date, or publication status. The search strategies for all databases are available in Supplemental Digital Content 1.

### Inclusion Criteria

#### Types of Studies

We included randomized controlled trials (RCTs) and excluded quasi-RCTs that used an inappropriate randomization method such as alternate allocation or allocation by birth date. Studies were excluded if they used the term “randomization” (随机) but failed to detail the randomization methods used. We included both parallel and crossover studies. Other study designs, such as *in vivo, in vitro*, case reports, and retrospective studies were excluded.

#### Types of Participants

We included studies involving patients diagnosed with TBI through medical or radiological examination, regardless of target symptoms, disease severity, sex, age, or race. We included all studies involving TBI patients, even if the diagnostic method of TBI was not clearly stated. We excluded studies that included participants with drug allergies or other serious medical conditions, such as cancer, liver disease, or kidney disease.

#### Types of Interventions

We included studies that used HM as a treatment intervention, regardless of which formulation of HM was used (e.g., decoction, tablets, capsules, pills, powders, and extracts); however, we only included studies in which HM was administered orally. We excluded studies that failed to detail the composition of the HM used, except when patent medicines were used whose composition could be found by searching the Internet. Studies comparing different types of HM were excluded. As control interventions, we included placebo, no treatment, and CT including surgery, medication, rehabilitation treatment, and psychotherapy for acute management and rehabilitation, which are baseline treatments for TBI. In the present study, acute management was defined as any treatment administered to stabilize the patients immediately after the injury (within 1 month). Rehabilitation was defined as any treatment of long-term impairments that aimed to restore to their previous level of health and was administered more than 1 month after injury (33). We included studies that combined HM with other therapies if the other therapies were used equally in both the treatment and control groups.

#### Types of Outcome Measures

The primary outcome measure was functional outcome, measured using the following validated scales: Barthel index (BI) (34), functional independence measurement (35), Fugl–Meyer assessment (36), and Glasgow Outcome Scale (GOS) (37). We also analyzed consciousness state measured using validated scales such as the GCS (38), with morbidity and mortality as primary outcome measures.

The secondary outcome measures were QoL, measured using validated assessment tools such as the 36-Item Short Form Health Survey (SF-36) (39), and adverse events (AEs), measured using the Treatment Emergent Symptom Scale (TESS) (40) or the incidence. We also analyzed the total effective rate (TER) as a secondary outcome; this is a non-validated outcome measure that is processed secondarily using certain evaluation criteria, such as improvement in clinical symptoms based on clinician ratings. In TER assessment, participants are generally classified as “cured” (痊愈), “markedly improved” (顯效), “improved” (有效), or “non-responsive” (無效) after treatment. The TER is calculated using the following formula: TER = *N1* + *N2* + *N3/N*, where *N1, N2, N3*, are the number of patients who are cured, markedly improved, and improved, respectively, while *N* is the total sample size. This outcome was considered a secondary outcome in this review as it lacks a unified standard and can be potentially heterogeneous.

### Study Selection

As previously reported, two researchers (B. Lee and C-Y Kwon) independently selected the studies according to the above inclusion criteria. After removing duplicates, we screened the titles and abstracts of the retrieved studies for relevance; we then evaluated the full texts of the selected studies for final inclusion. Any disagreement was resolved through discussion with the other authors.

### Data Extraction

Using a standardized data collection form in Excel 2007 (Microsoft, Redmond, WA, USA), two researchers (B. Lee and C-Y Kwon) independently extracted and double-checked the data from the included studies. Discrepancies were resolved through discussion with the other authors.

Using a predefined data collection form, we extracted information regarding the first author's name, publication year, country, institutional review board (IRB), informed consent, sample size, and number of dropouts, diagnostic criteria, participant details, intervention, comparisons, duration of intervention and follow-up, outcome measures, outcomes, and AEs. We also extracted details of the HM used, including the name, source, dosage form, and dosage of each medical substance, as well as the principles, rationale, and interpretation of the intervention in terms of the Consolidated Standards of Reporting Trials Extension for Chinese Herbal Medicine Formulas 2017 (41). If the data were insufficient or ambiguous, we contacted the corresponding authors of the included studies via e-mail to request additional information.

### Quality Assessment

As previously reported, two researchers (B. Lee and C-Y Kwon) independently evaluated the risk of bias of the included studies and the quality of evidence of the main findings. We resolved discrepancies through discussion with other researchers.

We assessed the methodological quality of the included studies using the Cochrane Collaboration's risk of bias tool (42). The following items were evaluated as either “low risk,” “unclear,” or “high risk”: (1) random sequence generation, (2) allocation concealment, (3) blinding of participants and personnel, (4) blinding of outcome assessment, (5) completeness of outcome data, (5) selective reporting, and (6) other biases. In particular, we assessed other bias categories with an emphasis on baseline imbalance between the treatment and control groups in terms of participant characteristics such as mean age, sex, or disease severity, because baseline imbalance in factors that are strongly related to outcome measures can cause bias when estimating the intervention effect.

The quality of evidence for each main finding was assessed using the Grading of Recommendations Assessment, Development, and Evaluation approach (43), which uses the online program GRADEpro (https://gradepro.org/). The following items were evaluated as either “very low,” “low,” “moderate,” or “high”: risk of bias, inconsistency, indirectness, and imprecision of the results, and probability of publication bias.

### Data Synthesis and Analysis

As previously described, we conducted descriptive analyses of the participants' details, interventions, and outcomes for all included studies. Using Review Manager version 5.3 software (Cochrane, London, UK), a meta-analysis was performed across studies that used the same types of intervention, comparison, and outcome measure. We pooled the dichotomous data using the risk ratio (RR) with 95% confidence intervals (CIs) and the continuous data using the mean difference (MD) with 95% CIs. We assessed clinical heterogeneity by comparing the distribution of important participant factors, such as age, sex, disease severity, and specific types of TBI, and we compared intervention factors such as co-interventions and control interventions among the included studies. Furthermore, statistical heterogeneity between the studies was assessed using both the chi-squared test and the *I*^2^ statistic; *I*^2^ ≥ 50% indicated substantial heterogeneity, while those ≥75% indicated high heterogeneity. In the meta-analyses, a random-effects model was used when the heterogeneity was significant (*I*^2^ ≥ 50%), while a fixed-effects model was used when the heterogeneity was not significant or when the number of studies included in the meta-analysis was <5, where estimates of inter-study variance have poor accuracy (44, 45). If the necessary data were available, we performed subgroup analyses to explain the heterogeneity or to assess whether the treatment effects varied between subgroups categorized according to the following criteria: (1) objective of interventions, such as acute management or rehabilitation, assessed in terms of time frame following injury; (2) severity of TBI, and (3) target symptoms, such as headache, dizziness, cognitive disorder, or mental disorder. To ascertain the robustness of the meta-analysis result, we conducted a sensitivity analyses by excluding (1) studies with a high risk of bias and (2) outliers that were numerically distant from the rest of the data.

### Reporting Bias

We assessed reporting biases, such as publication bias, using funnel plots if more than 10 studies were included in the meta-analysis.

## Results

### Study Description

**Figure 1:**
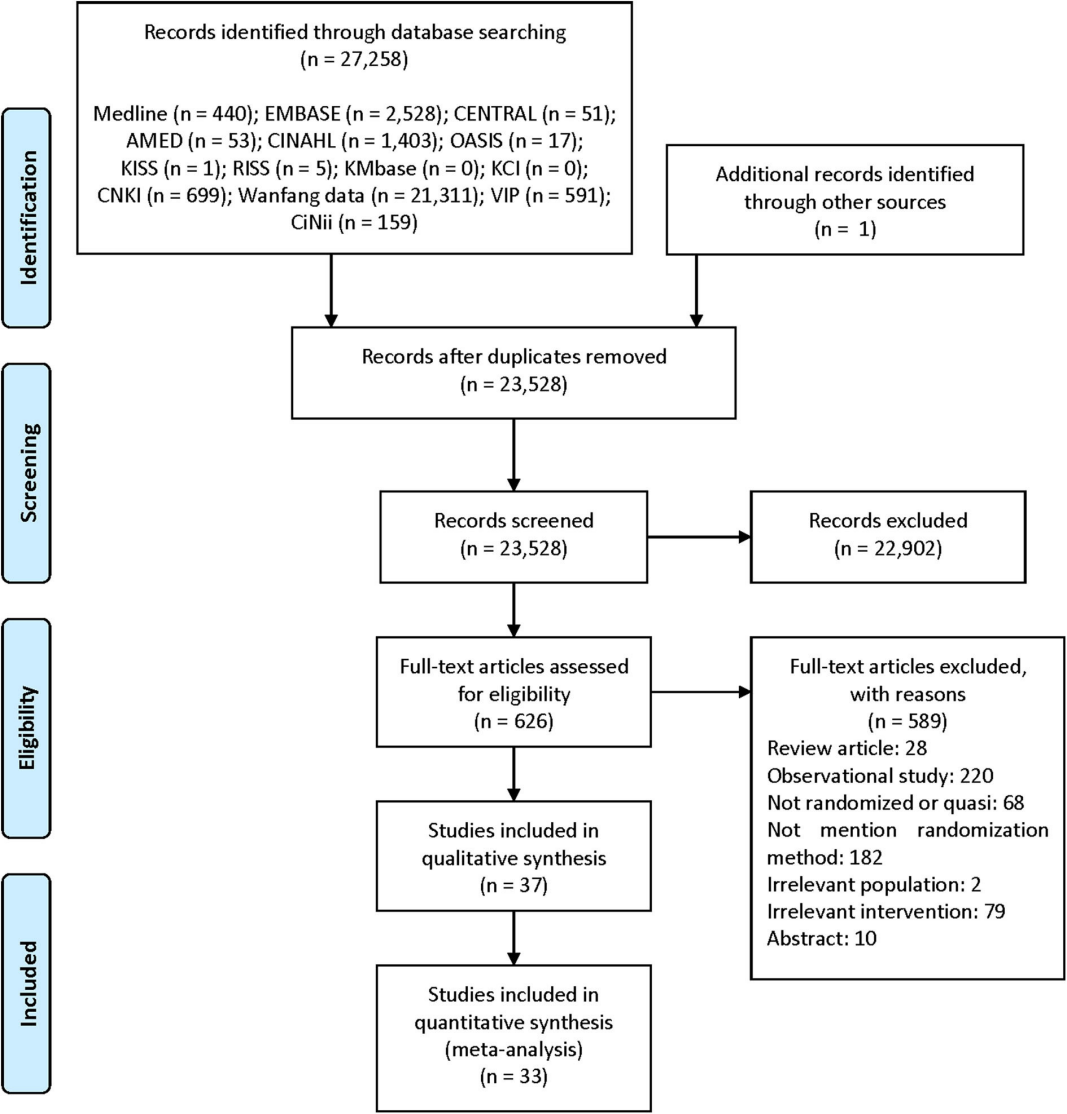
PRISMA flow diagram of the literature screening and selection processes Moher et al. (83). AMED, Allied and Complementary Medicine Database; CENTRAL, Cochrane Central Register of Controlled Trials; CINAHL, Cumulative Index to Nursing and Allied Health Literature; CNKI, China National Knowledge Infrastructure; KCI, Korea Citation Index; KISS, Koreanstudies Information Service System; KMbase, Korean Medical Database; OASIS, Oriental Medicine Advanced Searching Integrated System; RISS, Research Information Service System.

We identified 27,258 studies through database searching and one study from the references of the relevant studies. After removing duplicated studies, we considered 626 studies relevant after screening of the titles and abstracts. Among these, we finally included 37 studies with 3,374 participants (46–82) in the qualitative synthesis, and 33 studies with 3,000 participants (46–48, 50, 51, 53–59, 61–74, 76–82) in meta-analysis after screening of the full-text articles (Figure 1).

We have summarized the general characteristics of the included studies in Table 1. One study was conducted in New Zealand (46) and all others were conducted in China. The median sample size of the included studies was 80 participants (range: 30–300 participants), meanwhile, the median treatment period was 5 weeks (range: 3 days to 18 months). Eighteen studies (46, 49–51, 53, 57, 60, 63, 67, 68, 70–72, 76–80) reported the disease period of the participants; three of these (50, 68, 80) conducted treatment for acute management (from onset of injury to 1 month post-injury), while 11 (49, 51, 53, 57, 60, 63, 67, 70, 72, 77, 79) reported rehabilitation-focused treatment (>1 month post-injury). With regards to the specific symptoms treated, the included studies recruited patients with PCS (12 studies) (48, 49, 51, 54, 55, 57, 59, 60, 63, 78, 79, 82), mental disorder (four studies) (53, 62, 64, 66), cognitive dysfunction (four studies) (46, 61, 68, 76), epilepsy (four studies) (67, 70–72), mild TBI (four studies) (73–75, 80), headache (three studies) (50, 56, 81), dizziness (two studies) (47, 65), brain edema (one study) (58), and hydrocephalus (77).

**Table 1:** General characteristics of the included studies.

**Study ID**	**Sample size (included → analyzed)**	**Mean age (range; year)**	**Sex (M:F)**	**Disease period (mean interval between TBI and study enrollment)**	**Population**	**Pattern identification**	**(A) Experimental intervention**	**(B) Control intervention**	**Treatment period/F/U**	**Outcome**	**Results^+^**	**Adverse events**
(75)	88(44:44) → 88(44:44)	(A) 32.4 (14–53) (B) 33.4 (16–56)	(A) 31:13 (B) 34:10	NR	Mild TBI (No abnormalities in the CT, MRI, and nervous system examination)	NR	(B) + HM	Symptomatic treatment	5 d/NR	1. Clinical memory scale	1. (A)>(B)^*^ (all)^#^	NR
(73)	84(42:42) → 84(42:42)	(A) 36.8 ± 5.2 (15–55) (B) 37.2 ± 4.9 (16–58)	(A) 24:18 (B) 26:16	NR	Mild TBI–like symptoms (GCS ≥ 13, no abnormalities in CT)	NR	(B) + HM	Symptomatic treatment, bed rest, Nimodipine 30 mg tid	20 d/NR	1. Mean blood flow velocity of middle cerebral artery and basilar artery (Doppler flowmetry) 2. Clinical symptom relief time 3. TER (clinical symptom)	1. (A) < (B)^*^ (all) 2. (A) < (B)^*^ (all) 3. (A)>(B)^*^	None
(74)	80(40:40) → 80(40:40)	(A) 42 ± 9.8 (B) 40 ± 8.1	(A) 30:10 (B) 32:8	NR	Mild TBI (No abnormalities in vital sign and CT)	NR	HM, symptomatic treatment, bed rest	Symptomatic treatment, bed rest, Citicoline sodium 0.5 g plus 0.9% sodium chloride IV inj. qd	3–7 d/NR	1. TER (clinical symptom)	1. (A)>(B)+	NR
(47)	30(15:15) → 30(15:15)	(A) 42.3 ± 1.2 (B) 42.0 ± 1.9	(A) 10:5 (B) 9:6	NR	Dizziness	NR	HM	Nimodipine 30 mg tid	5 d/NR	1. TER (clinical symptom)	1. (A)>(B)^*^	NR
(50)	62(31:31) → 62(31:31)	(A) 38.7 ± 10.3 (20–62) (B) 38.3 ± 10.2 (19–61)	(A) 18:13 (B) 17:14	(A) 9.02 ± 2.16 d (2–16) (B) 9.57 ± 2.45 d (3–18)	Mild TBI induced headache	NR	(B) + HM	Symptomatic treatment, bed rest	NR/NR	1. TER (clinical symptom) 2. recurrence rate	1. (A)>(B)^*^ 2. (A) < (B)^*^	NR
(48)	156(78:78) → 156(78:78)	(A) 53.4 ± 8.2 (26–69) (B) 53.1 ± 8.2 (29–67)	(A) 25:53 (B) 24:54	NR	PCS (No abnormalities in the CT, MRI, CSF and nervous system examination)	Phlegm turbidity middle obstruction	HM	symptomatic treatment, psychotherapy	6 week/NR	1. TER (clinical symptom) 2. VAS (according to pattern identification)	1. (A)>(B)^*^ 2. (A) < (B)+ (all)	Mild transaminase elevation (A) 2, (B) 3; WBC elevation (A) 2, (B) 1; mild memory impairment (A) 3, (B) 4
(80)	80(40:40) → 80(40:40)	(A) 38.5 (12–60) (B) 40.5 (13–58)	(A) 22:18 (B) 26:14	(A) Mean 4.5 h (35 min−8 h) (B) Mean 5.2 h (45 min−7 h)	Mild TBI–like symptoms (GCS ≥ 13, no abnormalities in the CT)	NR	(B) + HM	Nimodipine 30 mg tid	20 d/NR	1. Mean blood flow velocity of middle cerebral artery and basilar artery (Doppler flowmetry) 2. TER (clinical symptom) 3. Number of people with clinical symptom relief	1. N.S (1 d after treatment), (A) < (B)^*^ (7, 14 and 20 d after treatment) 2. (A)>(B)^*^ 3. (A)>(B)^*^	None
(79)	60(30:30) → 60(30:30)	(A) 40.8 ± 10.3 (B) 41.3 ± 10.3	(A) 20:10 (B) 17:13	(A) 23.63 ± 13.58 mo (B) 24.65 ± 15.21 mo	PCS (No abnormalities in the CT, MRI, CSF, and nervous system examination)	Liver qi depression, blood deficiency, and spleen weakness	(B) + HM	Symptomatic treatment	6 wk/NR	1. TER (Rivermead post–concussion symptoms questionnaire score) 2. SF−36	1. (A)>(B)+ 2. (A)>(B)^*^ (vitality, social functioning, role limitations due to emotional problems and mental health), (A)>(B)+ (mental component summary), N.S (others)	None
(81)	300(150:150) → 300(150:150)	37.4	155:145	NR	Headache (No abnormalities in CT)	NR	HM	Analegics	2 mo/1 mo	1. TER (BRS−6) at 1 mo f/u 2. TER (headache)	1. (A)>(B)+ 2. (A)>(B)+	NR
(82)	124(62:62) → 124(62:62)	(A) 40.5 ± 5.5 (B) 41.2 ± 5.3	(A) 36:26 (B) 38:24	NR	PCS (No abnormalities in the CT and nervous system examination)	NR	(B) + HM	Piracetam 0.4 g tid, Oryzanol 10 mg tid, Nimodipine 20 mg tid, Nicergoline 20 mg tid, psychotherapy	4 wk/NR	1. TER (clinical symptom) 2. ADL 3. WMS	1. (A)>(B)+ 2. (A) < (B)+ 3. (A)>(B)+	None
(52)	120(60:60) → 120(60:60)	52.4 ± 10.4 (20–76)	82:38	NR	TBI (Mild TBI 53, SAH 29, brain contusion 18, subdural/epidural hematoma 20)	NR	(B) + HM	Conventional nutritional nerves and improved microcirculation therapy	NR/NR	1. Clinical symptom relief time 2. Coagulation items (plasmin prothrombin time, activity, activated paial prothrombin time, fibrinogen, thrombin time), platelet count, residual bleeding/total bleeding	1. (A) < (B)+ 2. (A) < (B)^*^ (residual bleeding/total bleeding), N.S (others)	NR
(55)	99(51:48) → 99(51:48)	(A) 45.6 ± 8.7 (18–66) (B) 43.96 ± 11.10 (17–65)	(A) 33:18 (B) 29:19	NR	PCS	Stasis and stagnation of qi and blood	(B) + HM	Psychotherapy, physical therapy, vitamin B, Oryzanol	2 wk/NR	1. TER (clinical symptom)	1. (A)>(B)+	None
(56)	96(48:48) → 96(48:48)	(A) 41 ± 5.8 (17–64) (B) 41 ± 4.6 (17–64)	(A) 30:18 (B) 29:19	NR	Headache (No abnormalities in CT or MRI)	NR	(B) + HM	Nimodipine 60 mg tid, Piracetam 0.8 g tid, symptomatic treatment	21 d/3 mo	1. TER (clinical symptom) 2. Headache symptom improvement time 3. recurrence rate (3 mo)	1. (A)>(B)^*^ 2. (A) < (B)^*^ 3. (A) < (B)^*^	(A) GI discomfort 1 (B) dizziness and mild nausea 2
(57)	60(30:30) → 60(30:30)	(A) 47.1 ± 6.4 (12–79) (B) 48.2 ± 11.3 (13–81)	(A) 18:12 (B) 17:13	(A) 12.03 ± 4.01 mo (6–18) (B) 12.15 ± 3.76 mo (6–18)	PCS (No abnormalities in CT, CSF and nervous system examination, no mental abnormalities)	Obstruction of clear orifices and blood stasis	(B) + HM	Symptomatic treatment, HBOT (once a day, total 30 times)	6–18 mo/NR	1. TCM syndrome score 2. Peak velocity and end–diastolic velocity of bilateral vertebral artery and basilar artery (Doppler flowmetry) 3. TER (clinical symptom, TCM syndrome score)	1. (A) < (B)+ 2. (A)>(B)^*^ (peak velocity of left vertebral artery and end–diastolic velocity of basilar artery), (A)>(B)+ (others) 3. (A)>(B)^*^	NR
(54)	100(60:40) → 100(60:40)	(A) 43.5 (B) 42.0	(A) 36:24 (B) 28:12	NR	PCS	NR	HM	Pyritinol hydrochloride 0.2 g tid	5 wk/NR	1. TER (clinical symptom)	1. (A)>(B)^*^	NR
(53)	80(40:40) → 80(40:40)	(A) 16–70 (B) 17–69	(A) 23:17 (B) 22:18	(A) 1–7 yr (B) 1–6.8 yr	Mental disorder (CCMD−3, HAMA≥14, HAMD≥17)	NR	(B) + HM	Fluoxetine 20 mg qd	8 wk/NR	1. TER (HAMD, HAMA, TESS) 2. HAMD 3. HAMA	1. (A)>(B)^*^ 2. (A) < (B)^*^ 3. (A) < (B)^*^	NR
(58)	40(20:20) → 40(20:20)	(A) 43.1 ± 17.7 (B) 47.8 ± 19.2	(A) 14:6 (B) 13:7	NR	Traumatic brain edema (GCS 9–15)	NR	HM	20% mannitol 125 ml IV inj.	14 d/1 mo	1. GCS 2. Intracranial pressure (mmH_2_O) 3. China stroke scale 4. Serum CRP concentration 5. Serum Na+ concentration 6. Serum K+ concentration 7. TER (TCM syndrome) 8. TER (clinical symptom) 9. TER (CT findings)	1. N.S 2. N.S 3. N.S 4. N.S 5. (A)>(B)+ 6. (A)>(B)+ 7. N.S 8. N.S 9. N.S	None
(75)	60(31:29) → 60(31:29)	(A) 35.8 ± 12.6 (B) 37.7 ± 19.9	(A) 19:12 (B) 18:11	(A) 15.10 ± 3.75 d (B) 16.50 ± 4.79 d	Cognitive dysfunction (3 < GCS ≤ 8)	NR	(B) + HM	Symptomatic treatment	54 d/NR	1. TER (Rancho Los Amigos levels of cognitive functioning scale) 2. Serum levels of NSE and S100β	1. (A)>(B)^*^ 2. (A) < (B)^*^ (all)	None
(77)	60(30:30) → 60(30:30)	(A) 47.1 ± 6.6 (35–66) (B) 46.7 ± 6.4 (37–64)	(A) 16:14 (B) 18:12	(A) 5.96 ± 0.81 mo (3–11) (B) 5.68 ± 0.76 mo (3–10)	Posttraumatic hydrocephalus	Phlegm and blood stasis obstructing the collaterals	(B) + HM	20% mannitol 125–250 ml IV inj. bid, acetazolamide 0.25 g bid–tid	15 d/1 mo	1. Serum levels of MBP, S100β, and p73 factor 2. NIHSS 3. BI 4. TCM syndrome scores 5. TER (clinical symptom and sign, degree of hydrocephalus, and TCM syndrome score) 6. Degree of hydrocephalus (f/u 1 mo)	1. (A) < (B)+ (MBP, S100β), N.S (p73 factor) 2. (A) < (B)+ 3. (A)>(B)+ 4. (A) < (B)+ 5. (A)>(B)^*^ 6. (A) < (B)^*^	None
(49)	80(40:40) → 80(40:40)	(A) 56.8 ± 12.3 (37–79) (B) 56.9 ± 10.8 (38–74)	(A) 21:19 (B) 22:18	(A) 1.2 ± 0.4 yr (0.4–1.8) (B) 1.1 ± 0.3 yr (0.3–1.6)	PCS (No abnormalities in CT and neurological examination)	NR	HM	Citicoline 0.5 g plus 10% glucose 200 ml IV inj. qd, Piracetam 0.8 g tid, Oryzanol 20 mg tid	2 mo/1 yr	1. TER (TCM syndrome)	1. (A)>(B)^*^	NR
(61)	70(35:35) → 70(35:35)	(A) 47.1 ± 14.3 (21–70) (B) 48.3 ± 15.3 (19–72)	(A) 26:9 (B) 28:7	NR	Cognitive dysfunction (MMSE <24, GCS 13–15)	NR	(B) + HM	Neurosurgery conventional treatment	1 mo/6 mo	1. MMSE 2. computer–aided cognitive measurement system	1. (A)>(B)^*^ (1 mo after treatment), (A) < (B)+ (f/u 6 mo) 2. (A)>(B)+ (1 mo, f/u 6 mo)	None
(51)	200(100:100) → 189(96:93)	(A) 34.2 ± 7.1 (B) 32.4 ± 6.7	(A) 64:32 (B) 64:32	(A) 7.55 ± 2.60 mo (B) 7.55 ± 3.17 mo	PCS (No abnormalities in the CT, MRI, CSF, and nervous system examination)	NR	(B) + HM	Psychological and behavioral therapy, symptomatic treatment, rehabilitation treatment	Until clinical symptoms disappeared for 2 wk or until 12 wk/NR	1. TER (clinical symptom) 2. Cure time	1. (A)>(B)+ 2. (A) < (B)^*^	(A) 4 (B) 2
(46)	78(36:42) → 53(25:28)	(A) 38.6 ± 14.1 (B) 38.4 ± 15.7	(A) 17:19 (B) 22:20	(A) Median 98 d (B) median 94.5 d	Cognitive dysfunction (cognitive failures questionnaire>30)	NR	HM	Placebo (dextrin and magnesium stearate)	6 mo/3 mo	1. CNS vital signs online neuropsychological test 2. Cognitive failures questionnaire 3. Rivermead postconcussionsymptom questionnaire 4. Quality of life 5. Hospital anxiety and depression scale 6. Modified fatigue impact scale 7. Extended GOS	1. (A) < (B)^*^ (complex attention, executive function), N.S (others) 2. N.S 3. N.S 4. N.S 5. N.S 6. N.S 7. N.S	(A) Headache 1, sore tongue 1, itchiness 1 (B) Difficulty sleeping 1, headache 1, itchiness 1, upset stomach 1, blood in urine 1
(68)	142(70:72) → 130(65:65)	38.6 (6–69)	74:56	13 ± 6 d (7–21)	Memory impairment (WMS <100, no aphasia)	NR	HM	Placebo (amylum)	4 wk/NR	1. memory quotient (WMS)	1. (A)>(B)+	(A) Nausea 2, diarrhea 2, mild hypotension 4 (B) none
(69)	112(56:56) → 112(56:56)	(A) 42.8 ± 5.1 (32–63) (B) 42.6 ± 5.1 (30–62)	(A) 36:20 (B) 33:23	NR	TBI	NR	HM	Placebo	8 wk/NR	1. Simple test for evaluating hand function 2. Fugi–Meyer assessment 3. Modified BI	1. (A)>(B)+ 2. (A)>(B)+ 3. (A)>(B)+	NR
(70)	68(34:34) → 68(34:34)	(A) 37.5 ± 2.6 (13–61) (B) 36.8 ± 2.4 (14–62)	(A) 19:15 (B) 18:16	(A) 4.5 ± 1.3 yr (2–7) (B) 4.3 ± 1.1 yr (1–8)	Epilepsy	NR	(B) + HM	Carbamazepine 5–20 mg/(kg·d)	NR/NR	1. TER (clinical symptom)	1. (A)>(B)^*^	(A) GI symptom 6, dizziness 3, rash 2, hair loss 3 (B) GI symptom 5, dizziness 4, rash 3, hair loss 2
(66)	40(20:20) → 40(20:20)	(A) 37.2 ± 3.5 (30–59) (B) 34.6 ± 5.7 (28–54)	(A) 12:8 (B) 14:6	NR	Mental disorder (CCMD-3)	NR	(B) + HM	Olanzapine 5–20 mg/d	8 wk/NR	1. PANSS 2. TESS	1. (A) < (B)^*^ 2. N.S	(A) GI discomfort 1, dizziness 1, dry mouth 1 (B) GI discomfort 2, nausea and vomiting 1, drowsiness 1, constipation 1, dry mouth 1
(67)	80(40:40) → 80(40:40)	(A) 64.2 ± 4.4 (19–88) (B) 63.9 ± 4.6 (19–87)	(A) 26:14 (B) 28:12	(A) 2.4 ± 0.4 mo (1–13) (B) 2.7 ± 0.3 mo (1–15)	Epilepsy	NR	HM	Sodium valproate sustained release tablets 500 mg bid	3 mo/1 mo	1. TER (clinical symptom) 2. Number of seizures	1. (A)>(B)^*^ 2. (A) < (B)^*^	None
(65)	96(48:48) → 96(48:48)	(A) 36 (22–68) (B) 40 (20–82)	(A) 31:17 (B) 35:13	NR	Dizziness	NR	HM	Flunarizine 5 mg bid	7–20 d/NR	1. TER (clinical symptom)	1. (A)>(B)+	NR
(64)	108(54:54) → 108(54:54)	(A) 58.0 ± 6.4 (B) 58.1 ± 6.9	(A) 32:22 (B) 30:24	NR	Mental disorder (CCMD-3)	NR	(B) + HM	Olanzapine 5–20 mg/d bid	8 wk/NR	1. PANSS 2. TESS 3. Brief psychiatric rating scale 4. GQOLI-74	1. (A) < (B)^*^ 2. N.S 3. (A) < (B)^*^ 4. (A)>(B)^*^ (body health, psychological conditions, social function), N.S (others)	(A) Nausea and vomiting 2, dizziness 1, GI discomfort 1, dry mouth 1 (B) Nausea and vomiting 3, GI discomfort 2, drowsiness 1, constipation 1
(78)	78(43:35) → 78(43:35)	(A) 39.2 ± 5.0 (18–58) (B) 38.7 ± 6.2 (20–63)	(A) 18:25 (B) 11:24	(A) 14.4 ± 4.5 mo (B) 16.8 ± 3.7 mo	PCS	NR	HM	Oryzanol tid	2 wk/NR	1. TER (clinical symptom)	1. (A)>(B)^*^ (all)	NR
(60)	86(43:43) → 86(43:43)	(A) 52.3 ± 10.2 (34–68) (B) 53.1 ± 10.2 (32–67)	(A) 22:21 (B) 19:24	(A) 20.59 ± 4.12 mo (2–36) (B) 18.26 ± 4.52 mo (3–36)	PCS	Blood stasis affecting the clear orifices	(B) + HM	HBOT (once a day, 5 times per week)	4 wk/NR	1. TCM syndrome score 2. TER (TCM syndrome score) 3. NIHSS 4. Mean blood flow velocity of bilateral vertebral artery and basilar artery (Doppler flowmetry)	1. (A) < (B)^*^ 2. (A)>(B)^*^ 3. (A) < (B)^*^ 4. (A) < (B)^*^ (all)	NR
(59)	50(25:25) → 50(25:25)	(A) 45.2 ± 1.0 (30–60) (B) 46.2 ± 1.3 (31–60)	(A) 13:12 (B) 14:11	NR	PCS	NR	HM	Oryzanol 20 mg tid	NR/NR	1. TER (clinical symptom) 2. Symptom improvement time 3. Hospitalization time	1. (A)>(B)^*^ 2. (A) < (B)^*^ 3. (A) < (B)^*^	NR
(62)	48(24:24) → 48(24:24)	(A) 34.5 ± 5.2 (28–52) (B) 35.1 ± 5.7 (30–54)	(A) 14:10 (B) 16:8	NR	Mental disorder	NR	(B) + HM	Olanzapine 5–20 mg/d	8 wk/NR	1. PANSS 2. TESS 3. TER (clinical symptom)	1. (A) < (B)+ 2. (A) < (B)+ 3. (A)>(B)^*^	(A) GI discomfort 1, dry mouth 1 (B) GI discomfort 2, constipation 2, dry mouth 2, drowsiness 2
(71)	60(30:30) → 60(30:30)	(A) 31.5 ± 15.5 (B) 30.5 ± 13.7	(A) 26:4 (B) 25:5	(A) 6.2 ± 3.10 yr (B) 6.4 ± 2.9 yr	Epilepsy	NR	(B) + HM	Carbamazepine 0.1 g tid, γ-aminobutyric acid 1.5 g tid	2 mo/0.5 yr	1. TER (clinical symptom)	1. No statistical analysis	(A) rash 2, drowsiness 2, nausea 1 (B) leukopenia 4, rash 4, drowsiness 5, nausea 3
(72)	79(41:38) → 79(41:38)	(A) 28–65 (B) 25–63	(A) 28:13 (B) 26:12	(A) NR (1 mo−3 yr) (B) NR (1 mo−2.5 yr)	Epilepsy	NR	(B) + HM	Sodium valproate sustained-release tablets 500 g bid	3 mo/NR	1. TER (clinical symptom)	1. (A)>(B)^*^	NR
(63)	120(60:60) → 120(60:60)	(A) 50.6 ± 8.2 (B) 48.7 ± 9.1	(A) 36:24 (B) 34:26	(A) 12.47 ± 4.64 mo (B) 12.62 ± 4.96 mo	PCS (No abnormalities in CT)	blood stasis obstructing clear orifices and blood stasis	(B) + HM	Diclofenac sodium sustained release capsule 25 mg bid, Piracetam 0.8 g tid, Oryzanol 20 mg tid, HBOT (once a day)	1 mo/NR	1. TCM syndrome score 2. Mean blood flow velocity of bilateral vertebral artery and basilar artery (Doppler flowmetry) 3. TER (clinical symptom, TCM syndrome score)	1. (A) < (B)+ 2. (A)>(B)^*^ (all) 3. (A)>(B)^*^	None

“*” and “+” *mean significant differences between two groups, p <0.05 and p <0.01, respectively. “N.S” means no significant difference between two groups, p > 0.05*.

# *“all” means that all of the subscales in the outcome measurement tool were improved*.

*ADL, activities of daily living; BI, barthel index; BRS-6, 6-point behavioral rating scale; CCMD, Chinese classification of mental disorders; CNS, central nervous system; CRP, C-reactive protein; CSF, cerebrospinal fluid; CT, computed tomography; F/U, follow up; GCS, Glasgow coma scale; GI, gastrointestinal; GOS, Glasgow outcome scale; GQOLI-74, generic quality of life inventory-74; HAMA, Hamilton anxiety rating scale; HAMD, Hamilton depression rating scale; HBOT, hyperbaric oxygen therapy; HM, herbal medicine; MBP, myelin basic protein; MMSE, mini-mental state examination; MRI, magnetic resonance imaging; NIHSS, national institute of health stroke scale; NR, not reported; NSE, neuron-specific enolase; PANSS, positive and negative symptoms scale; PCS, post-concussion syndrome; SAH, subarachnoid hemorrhage; SF-36, 36-item short form survey; TBI, traumatic brain injury; TCM, traditional Chinese medicine; TER, total effective rate; TESS, treatment emergent symptom scale; VAS, visual analog scale; WBC, white blood cell; WMS, Wechsler memory scale*.

Seven studies recruited participants based on pattern identification (an approach of some East-Asian traditional medicines, including traditional Chinese medicine, which enables individual treatment by categorizing the signs and symptoms of patients into a series of syndrome concepts): five based on “blood stasis” (55, 57, 60, 63, 77), two on “phlegm” (48, 77), and one on “liver qi depression, blood deficiency, and spleen weakness” (79). Eleven studies compared HM with CT (47–49, 54, 58, 59, 65, 67, 74, 78, 81), three compared HM with a placebo (46, 68, 69), and 23 compared HM plus CT with CT alone (50–53, 55–57, 61, 70, 73, 75–77, 79, 80, 82). The CTs included symptomatic treatment, routine rehabilitation care, psychotherapy, and Western medication. Nine studies (46, 49, 56, 58, 61, 67, 71, 77, 81) conducted follow-up after treatment, with the range of follow-up periods being 1 month to 1 year. Various outcome measures were used depending on the target population, with the most frequently used outcome being TER, assessed in 29 studies (46–51, 53–60, 62, 63, 65, 67, 70, 72–74, 76–82). Ten studies (46, 50, 52, 56, 57, 59, 64, 69, 76, 77) reported IRB approval, and 20 (46, 48, 50–52, 56–61, 63, 64, 66, 69, 76–79, 82) reported that they had received consent from the participants.

The included studies used a variety of HMs, with the most common being *Xuefuzhuyu* decoction (six studies) (50, 60, 62, 66, 67, 72), followed by the patented drug *Yangxue Qingnao* granules (four studies) (68, 73, 80, 82). In total, 89 different herbs were used in the included studies, with the most frequently used being *Cnidii Rhizoma* (27 studies), followed by *Angelicae Gigantis Radix* (25 studies), *Persicae Semen* (19 studies), *Carthami Flos* (17 studies), *Bupleuri Radix* (16 studies), *Paeoniae Radix Rubra* (16 studies), and *Acori Graminei Rhizoma* (15 studies) (Supplemental Digital Content 2).

### Risk of Bias

**Figure 2:**
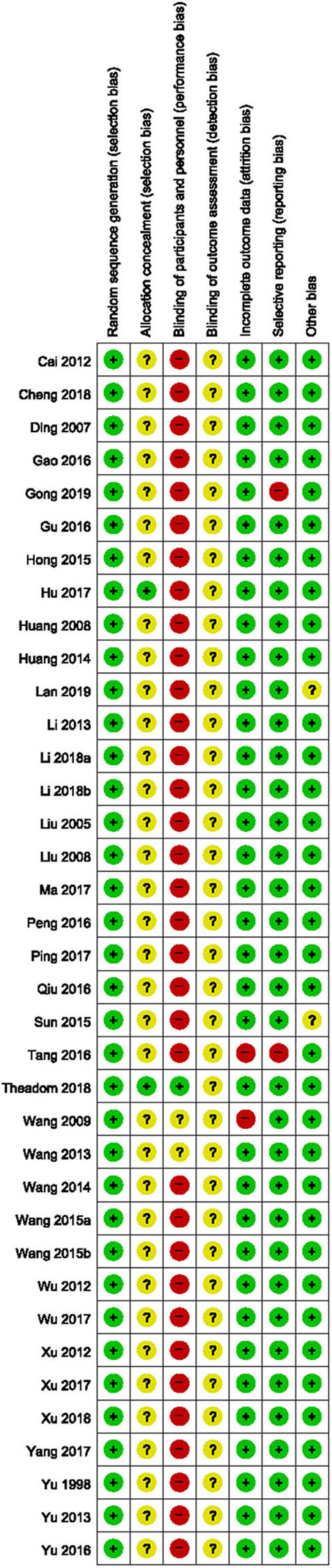
Risk of bias summary for all included studies. Low, unclear, and high risk, respectively, are represented with the following symbols: “+”, “?”, and “–”.

**Figure 3:**
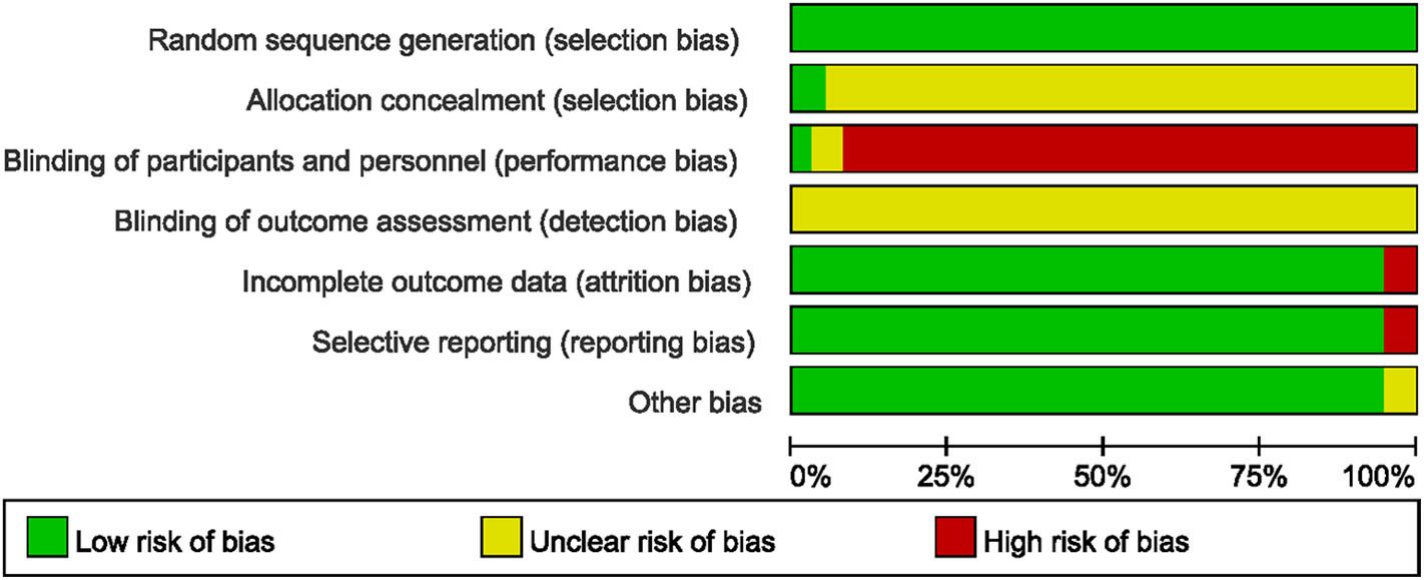
Risk of bias graph for all included studies.

All the included studies reported appropriate random sequence generation methods; however, only two used a sealed opaque envelope (79) or independent allocation manager (46) to conceal allocation. Only one study (46) appropriately blinded both the participants and personnel, and two studies (68, 69) used placebo drugs as a control intervention but did not report appropriate blinding of personnel. None of the included studies reported blinding of the outcome assessor. Two studies (51, 68) that performed per-protocol analysis were assessed as having a high risk of attrition bias, while two (50, 51) that reported only TER, a secondary processed outcome without the raw data, were assessed as having a high risk of reporting bias. Thirty-five studies (46–51, 53–60, 62–82) reported no significant baseline difference in demographic data between the two groups, and were rated as having low risk of bias in the other potential sources of bias domains (Figures 2, 3).

### HM vs. CT

#### Effectiveness

Eleven studies (47–49, 54, 58, 59, 65, 67, 74, 78, 81) were included in the comparison of effectiveness: five (48, 49, 54, 59, 78) were conducted on patients with PCS, (47, 65) two on patients with dizziness, one each on patients with headache (81), epilepsy (67), mild TBI (74), and traumatic brain edema (58). Although there were no differences in the functional outcomes and states of consciousness between two groups, HM group showed significantly better outcomes in TER based on clinical symptoms, symptom improvement time, and duration of hospitalization.

**Figure 4:**
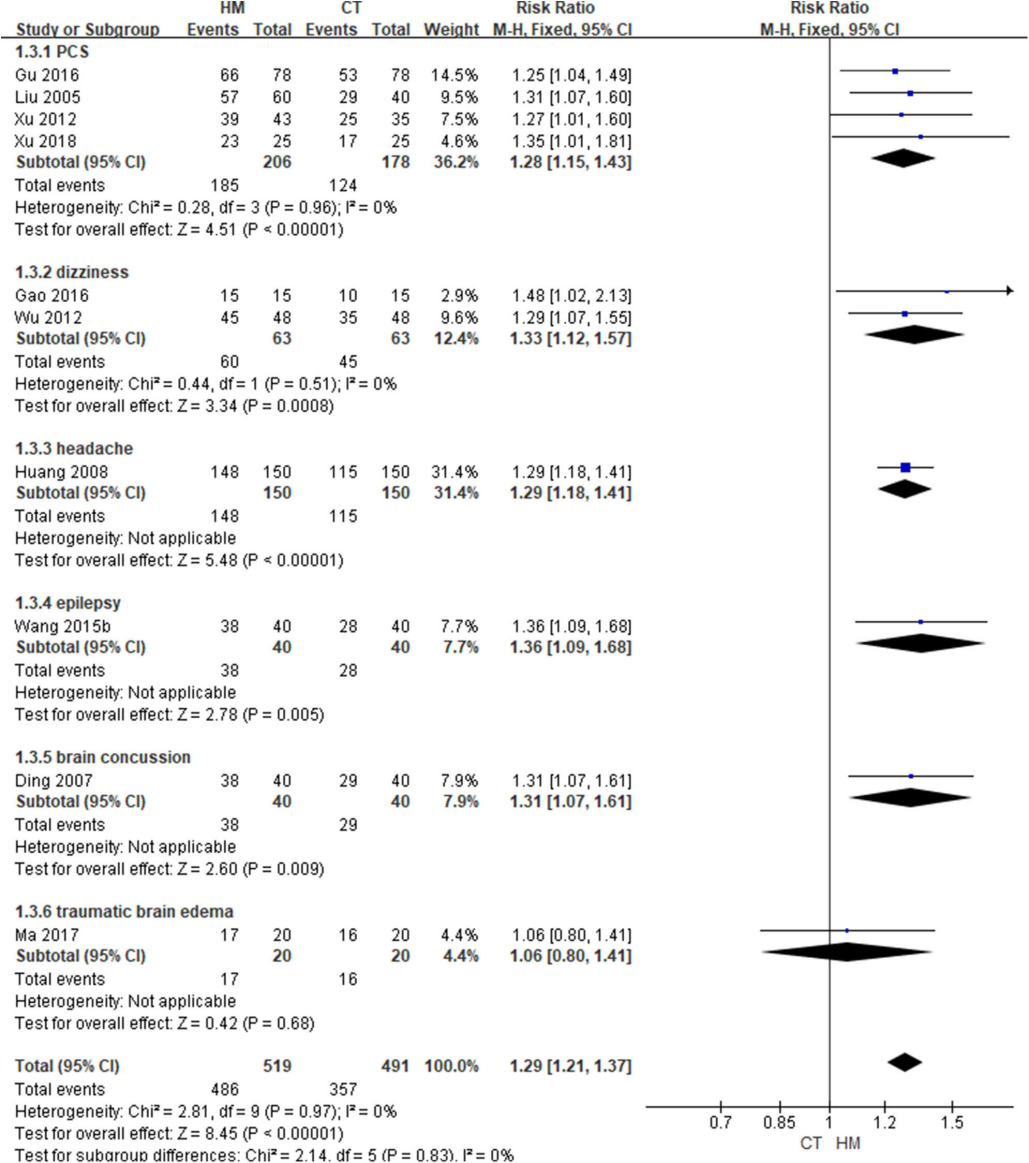
Total Effective rate based on clinical symptoms (Comparison of herbal medicine vs. conventional treatment).

In one study involving traumatic brain edema (58), the groups did not differ in terms of functional outcome, as measured using the GOS, after 1 month of post-intervention follow-up (MD: 0.10, 95% CI: −0.13 to 0.33), nor did they differ in terms of consciousness state, measured using the GCS after 14 days of intervention (MD: 0.05, 95% CI: −0.12–0.22). In addition, the two groups did not differ in terms of intracranial pressure or neurological function, measured using the China stroke scale after treatment. However, in 10 studies, the TER based on clinical symptoms was significantly improved in the HM group (RR: 1.29, 95% CI: 1.21–1.37, *I*^2^ = 0%). In a subgroup analysis based on the target symptoms of TBI, the HM group showed significantly better outcomes in patients with PCS, dizziness, headache, epilepsy, and mild TBI of all causes except traumatic brain edema (Table 2; Figure 4) (Supplemental Digital Content 3).

**Table 2:** Summary of findings in all comparisons.

**Outcomes**		**No. participants (RCTs)**	**Anticipated absolute effects (95% CI)**		**Relative effect (95% CI)**	**Quality of evidence (GRADE)**	**Comments**
			**Risk with control group**	**Risk with treatment group**			
**Herbal medicine vs. conventional treatment**							
GOS	Total (traumatic brain edema)	40 (1)	–	MD 0.1 higher (0.13 lower to 0.33 higher)	–	⊕○○○ VERY LOW	Risk of bias (−1) Imprecision (−2)
GCS	Total (traumatic brain edema)	40 (1)	–	MD 0.05 higher (0.12 lower to 0.22 higher)	–	⊕○○○ VERY LOW	Risk of bias (−1) Imprecision (−2)
TER (clinical symptom)	Total	1,010 (10)	727 per 1,000	938 per 1,000 (880–996)	RR 1.29 (1.21–1.37)	⊕⊕○○ LOW	Risk of bias (−1) Indirectness (−1)
Subgroup (target symptom)	PCS	384 (4)	697 per 1,000	892 per 1,000 (801–996)	RR 1.28 (1.15–1.43)	⊕⊕○○ LOW	Risk of bias (−1) Indirectness (−1)
	Dizziness	126 (2)	714 per 1,000	950 per 1,000 (800–1,000)	RR 1.33 (1.12–1.57)	⊕⊕○○ LOW	Risk of bias (−1) Indirectness (−1)
	Headache	300 (1)	767 per 1,000	989 per 1,000 (905–1,000)	RR 1.29 (1.18–1.41)	⊕⊕○○ LOW	Risk of bias (−1) Indirectness (−1)
	Epilepsy	80 (1)	700 per 1,000	952 per 1,000 (763–1,000)	RR 1.36 (1.09–1.68)	⊕○○○ VERY LOW	Risk of bias (−1) Indirectness (−1) Imprecision (−1)
	Mild TBI	80 (1)	725 per 1,000	950 per 1,000 (776–1,000)	RR 1.31 (1.07–1.61)	⊕○○○ VERY LOW	Risk of bias (−1) Indirectness (−1) Imprecision (−1)
	Traumatic brain edema	40 (1)	800 per 1,000	848 per 1,000 (640–1,000)	RR 1.06 (0.80–1.41)	⊕○○○ VERY LOW	Risk of bias (−1) Indirectness (−1) Imprecision (−1)
AE	Total	276 (3)	58 per 1,000	51 per 1,000 (19–133)	RR 0.88 (0.33–2.30)	⊕○○○ VERY LOW	Risk of bias (−1) Imprecision (−2)
Subgroup (target symptom)	PCS	156 (1)	103 per 1,000	90 per 1,000 (34–236)	RR 0.88 (0.33–2.30)	⊕○○○ VERY LOW	Risk of bias (−1) Imprecision (−2)
	Epilepsy	80 (1)	0 per 1,000	0 per 1,000 (0–0)	Not estimable	⊕⊕○○ LOW	Risk of bias (−1) Imprecision (−1)
	Traumatic brain edema	40 (1)	0 per 1,000	0 per 1,000 (0–0)	Not estimable	⊕⊕○○ LOW	Risk of bias (−1) Imprecision (−1)
**Herbal medicine vs. placebo**							
Fugl–Meyer assessment	Total	112 (1)	–	MD 9.63 higher (8.21–11.05 higher)	–	⊕⊕⊕○ MODERATE	Risk of bias (−1)
Modified BI	Total	112 (1)	–	MD 18.54 higher (17.27–19.81 higher)	–	⊕⊕⊕○ MODERATE	Risk of bias (−1)
GOS	Total (cognitive dysfunction)	53 (1)	–	MD 0 (4.17 lower−4.17 higher)	–	⊕⊕○○ LOW	Imprecision (−2)
QoL	Total (cognitive dysfunction)	53 (1)	–	MD 1.91 higher (9.58 lower−13.40 higher)	–	⊕⊕○○ LOW	Imprecision (−2)
AE	Total (cognitive dysfunction)	208 (2)	47 per 1,000	107 per 1,000 (39–295)	RR 2.29 (0.83–6.32)	⊕○○○ VERY LOW	Risk of bias (−1) Inconsistency (−1) Imprecision (−2)
**Herbal medicine plus conventional treatment vs. conventional treatment alone**							
ADL	Total (PCS)	124 (1)	–	MD 3.30 lower (5.04–1.56 lower)	–	⊕⊕⊕○ MODERATE	Risk of bias (−1)
BI	Total (posttraumatic hydrocephalus)	60 (1)	–	MD 11.14 higher (5.43–16.85 higher)	–	⊕⊕○○ LOW	Risk of bias (−1) Imprecision (−1)
SF−36 (physical component summary)	Total (PCS)	60 (1)	–	MD 3.84 higher (13.27 lower−20.95 higher)	–	⊕○○○ VERY LOW	Risk of bias (−1) Imprecision (−2)
SF−36 (mental component summary)	Total (PCS)	60 (1)	–	MD 36.51 higher (13.76–59.26 higher)	–	⊕⊕○○ LOW	Risk of bias (−1) Imprecision (−1)
GQOLI−74 (physical health)	Total (mental disorder)	108 (1)	–	MD 11.68 higher (9.11–14.25 higher)	–	⊕⊕⊕○ MODERATE	Risk of bias (−1)
GQOLI−74 (psychological health)	Total (mental disorder)	108 (1)	–	MD 24.41 higher (21.94–26.88 higher)	–	⊕⊕⊕○ MODERATE	Risk of bias (−1)
GQOLI−74 (social functional status)	Total (mental disorder)	108 (1)	–	MD 13.67 higher (11.14–16.20 higher)	–	⊕⊕⊕○ MODERATE	Risk of bias (−1)
GQOLI−74 (living condition)	Total (mental disorder)	108 (1)	–	MD 1.01 higher (1.52 lower−3.54 higher)	–	⊕⊕○○ LOW	Risk of bias (−1) Imprecision (−1)
TER (clinical symptom)	Total	1,429 (17)	762 per 1,000	922 per 1,000 (883–967)	RR 1.21 (1.16–1.27)	⊕⊕○○ LOW	Risk of bias (−1) Indirectness (−1)
Subgroup (target symptom)	PCS	652 (6)	774 per 1,000	944 per 1,000 (882–1,000)	RR 1.22 (1.14–1.30)	⊕⊕○○ LOW	Risk of bias (−1) Indirectness (−1)
	Mental disorder	128 (2)	781 per 1,000	938 per 1,000 (813–1,000)	RR 1.20 (1.04–1.39)	⊕○○○ VERY LOW	Risk of bias (−1) Indirectness (−1) Imprecision (−1)
	Cognitive dysfunction	60 (1)	862 per 1,000	940 per 1,000 (784–1,000)	RR 1.09 (0.91–1.29)	⊕○○○ VERY LOW	Risk of bias (−1) Indirectness (−1) Imprecision (−2)
	Headache	158 (2)	747 per 1,000	926 per 1,000 (799–1,000)	RR 1.24 (1.07–1.43)	⊕⊕○○ LOW	Risk of bias (−1) Indirectness (−1)
	Epilepsy	207 (3)	735 per 1,000	882 per 1,000 (772–1,000)	RR 1.20 (1.05–1.38)	⊕⊕○○ LOW	Risk of bias (−1) Indirectness (−1)
	Posttraumatic hydrocephalus	60 (1)	733 per 1,000	865 per 1,000 (667–1,000)	RR 1.18 (0.91–1.53)	⊕○○○ VERY LOW	Risk of bias (−1) Indirectness (−1) Imprecision (−2)
	Mild TBI–like symptoms	164 (2)	720 per 1,000	899 per 1,000 (777–1,000)	RR 1.25 (1.08–1.46)	⊕⊕○○ LOW	Risk of bias (−1) Indirectness (−1)
AE	Total	1,386 (16)	78 per 1,000	50 per 1,000 (34–73)	RR 0.64 (0.44–0.93)	⊕○○○ VERY LOW	Risk of bias (−1) Imprecision (−2)
Subgroup (target symptom)	PCS	592 (5)	7 per 1,000	13 per 1,000 (2–70)	RR 1.94 (0.36–10.33)	⊕○○○ VERY LOW	Risk of bias (−1) Imprecision (−2)
	Mental disorder	216 (3)	178 per 1,000	96 per 1,000 (48–192)	RR 0.54 (0.27–1.08)	⊕○○○ VERY LOW	Risk of bias (−1) Imprecision (−2)
	Cognitive dysfunction	130 (2)	0 per 1,000	0 per 1,000 (0–0)	Not estimable	⊕⊕○○ LOW	Risk of bias (−1) Imprecision (−1)
	headache	96 (1)	42 per 1,000	21 per 1,000 (2–222)	RR 0.50 (0.05–5.33)	⊕○○○ VERY LOW	Risk of bias (−1) Imprecision (−2)
	Epilepsy	128 (2)	469 per 1,000	295 per 1,000 (188–473)	RR 0.63 (0.40–1.01)	⊕○○○ VERY LOW	Risk of bias (−1) Inconsistency (−2) Imprecision (−2)
	Posttraumatic hydrocephalus	60 (1)	0 per 1,000	0 per 1,000 (0–0)	Not estimable	⊕⊕○○ LOW	Risk of bias (−1) Imprecision (−1)
	Mild TBI–like symptoms	164 (2)	0 per 1,000	0 per 1,000 (0–0)	Not estimable	⊕⊕○○ LOW	Risk of bias (−1) Imprecision (−1)
TESS	Total (mental disorder)	196 (3)	–	MD 1.05 lower (1.46–0.64 lower)	–	⊕⊕⊕○ MODERATE	Risk of bias (−1)

*ADL, activities of daily living; AE, adverse event; BI, Barthel index; CI, confidence interval; CT, conventional treatment; GCS, Glasgow coma scale; GOS, Glasgow outcome scale; GQOLI-74, generic quality of life inventory-74; GRADE, grading of recommendations assessment, development, and evaluation; MD, mean difference; PCS, post-concussion syndrome; QoL, quality of life; RCT, randomized controlled trial; RR, risk ratio; SF-36, 36-item short forms*.

In a study by Xu et al. (59), when HM was administered to patients with PCS, the symptom improvement time and hospitalization time were significantly shorter than in the CT group (*P* < 0.05, all). Wang and Tian (67) reported that, when HM was administered to patients of epilepsy, the number of seizures was significantly lower than in the CT group (*P* < 0.05).

#### Safety

Three studies reported AEs during the intervention, and a meta-analysis of these showed no difference in the incidence of AEs between the two groups (RR: 0.88, 95% CI: 0.33–2.30; Table 2) (Supplemental Digital Content 3).

### HM vs. Placebo

#### Efficacy

Three studies (46, 68, 69) compared HM with a placebo. Two of these (46, 68) were conducted on patients with cognitive dysfunction, while the other one (69) did not include participants with specific symptoms. Collectively, the functional outcomes showed inconsistent results between studies, and there was no significant difference in QoL between two groups. However, memory impairment was improved more in the HM group.

In a study by Wang (69), the HM group showed improved functional outcomes, as assessed using the Fugl–Meyer assessment (MD: 9.63, 95% CI: 8.21–11.05) and modified BI (MD: 18.54, 95% CI: 17.27–19.81), after 8 weeks of treatment. Additionally, hand function in the HM group was significantly better than in the placebo group (*P* < 0.01). After patients with cognitive dysfunction were treated ifor 6 months (46), physical disability was measured using the GOS and QoL measured by the QoL after brain injury scale showed no significant differences between the two groups (GOS: MD, 0.00; 95% CI: −4.17 to 4.17; QoL after brain injury scale: MD, 1.91; 95% CI: −9.58 to 13.40; Table 2) (Supplemental Digital Content 3). In addition, after intervention, there were no significant differences between the groups in terms of neurobehavioral sequelae, mood, or fatigue. However, complex attention and executive functioning in the HM group were significantly better than in the placebo group (*P* < 0.05). In a study by Wang et al. (68) involving patients with memory impairment, the HM group showed significantly better memory quotient, measured using the Wechsler Memory Scale, than the placebo group after 4 weeks of treatment (*P* < 0.01). The results of sensitivity analysis by excluding low quality studies (that had 4 or less low risk of bias on the seven domains of the risk of bias tool) were consistent in GOS and QoL (Supplemental Digital Content 4).

#### Safety

Two studies (46, 68) recruiting patients with cognitive dysfunction reported AEs during the treatment period. There was no difference in the incidence of AEs between the two groups (RR: 2.29, 95% CI: 0.83–6.32, and *I*^2^ = 79%; Table 2; Figure 4) (Supplemental Digital Content 3), nor was there any difference between the two groups in a sensitivity analysis that excluded studies with a high risk of bias (Supplemental Digital Content 4).

### HM Plus CT vs. CT Alone

#### Effectiveness

Twenty-three studies (50–53, 55–57, 61, 70, 73, 75–77, 79, 80, 82) compared effectiveness between HM plus CT and CT alone. Seven of these (51, 55, 57, 60, 63, 79, 82) were conducted on patients with PCS, four (53, 62, 64, 66) on patients with mental disorder, three on patients with epilepsy (70–72), three on patients with mild TBI (73, 75, 80), two on patients with cognitive dysfunction (61, 76), two on patients with headache (50, 56), and one each on patients with hydrocephalus (77) and TBI (52). In summary, the function and TER of various symptoms were significantly improved when HM was added to CT. However, there were inconsistent results in QoL between studies.

Huang and Li (82) conducted 4 weeks of treatment in patients with PCS; they found that activities of daily living were significantly better in the HM plus CT group than in the CT alone group (MD: −3.30, 95% CI: −5.04 to −1.56). Ping (77) conducted 15 days of treatment in patients with post-traumatic hydrocephalus; their results showed that functional outcomes, as measured using BI, were significantly better in the HM group (MD: 11.14, 95% CI: 5.43–16.85) (Table 2) (Supplemental Digital Content 3). When HM was added to the CT, there was a significant difference in neurological function after treatment compared to that with CT alone, as measured using the National Institute of Health Stroke Scale (NIHSS) (*P* < 0.01), and degree of hydrocephalus differed significantly between the groups after 1 month of post-intervention follow-up (*P* < 0.05) (77).

**Figure 5:**
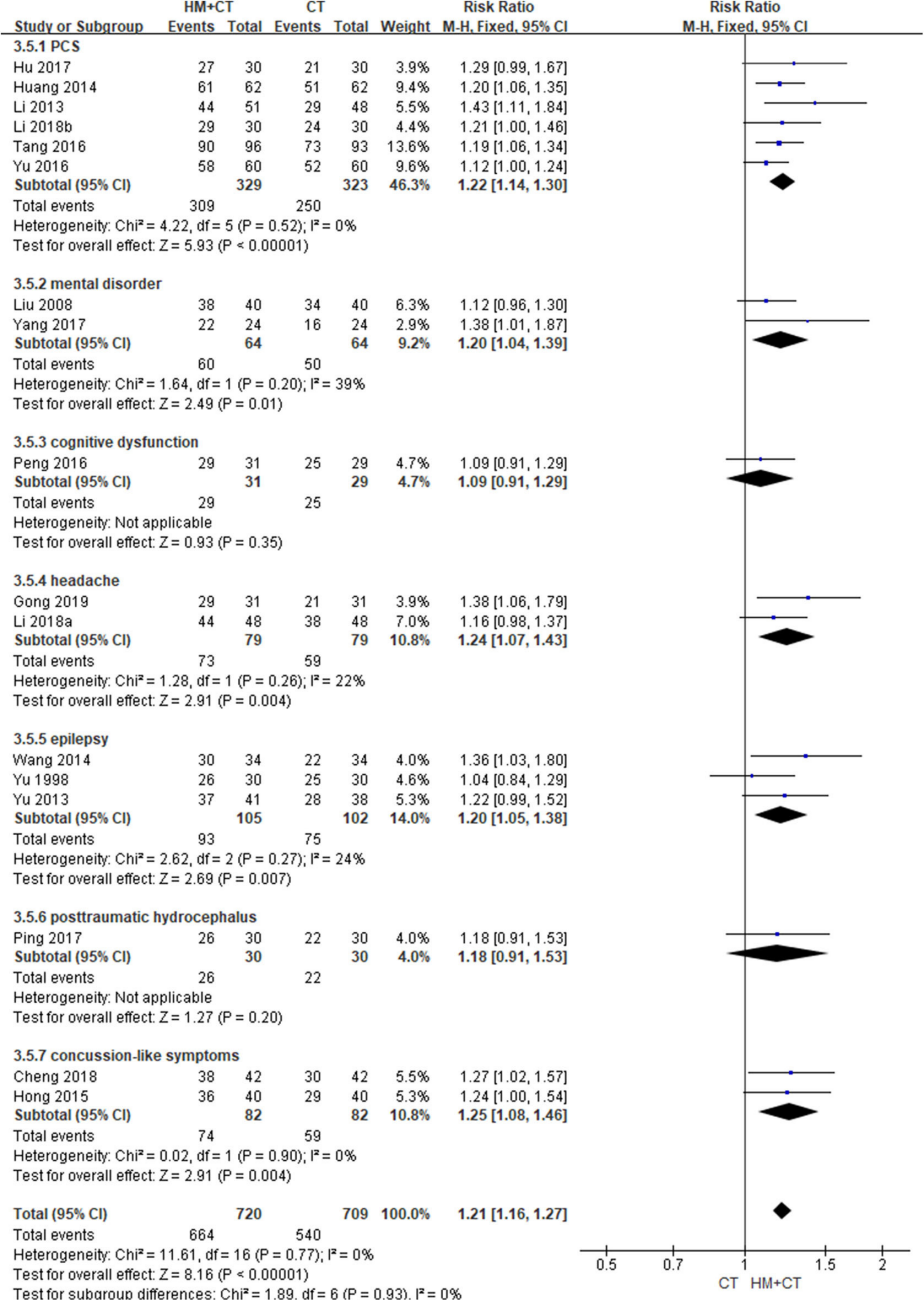
Total effective rate based on clinical symptoms (Comparison of herbal medicine combined with conventional treatment vs. conventional treatment alone).

Two studies (64, 79) reported the QoL of patients after treatment. One (79) showed that patients with PCS treated using HM had significantly better mental component summary score, as measured using the SF-36 scale, than the CT alone group after 6 weeks of treatment (MD: 36.51, 95% CI: 13.76–59.26). However, there was no difference in physical component summary score (MD: 3.84, 95% CI: −13.27–20.95). Another study (64) treated patients with mental disorder for 8 weeks. The HM group showed significantly better scores in the areas of physical health, psychological health, and social functional status domain, measured using the generic QoL inventory 74. However, there was no difference between the groups in terms of living condition (physical health: MD, 11.68, 95% CI, 9.11–14.25; psychological health: MD, 24.41, 95% CI, 21.94–26.88; social functional status: MD, 13.67, 95% CI, 11.14–16.20; living condition: MD, 1.01, 95% CI,−1.52–3.54). The HM group showed significantly better TER, based on clinical symptoms (17 studies; RR: 1.21, 95% CI: 1.16–1.27, *I*^2^ = 0%) (Figure 5). In a subgroup analysis according to target symptoms of TBI, there were significant differences in PCS, mental disorder, headache, epilepsy, and mild TBI-like symptoms, but not in cognitive dysfunction or post-traumatic hydrocephalus (Table 2) (Supplemental Digital Content 3). However, a sensitivity analysis that excluded studies with a high risk of bias showed no difference in TER based on clinical symptoms between the two groups (Supplemental Digital Content 4).

When HM plus CT was administered to treat patients with PCS, neurological function, as measured using the NIHSS, was better than when CT alone was used (*P* < 0.05) (60), and cure time was significant shorter in the combination group (*P* < 0.05) (51). In patients with mental disorder after TBI, symptoms of depression (53), anxiety (53), and schizophrenia (62, 64, 66) were significantly better in the combination group than in the CT alone group (*P* < 0.05 in all cases). Furthermore, when HM plus CT was administered, cognitive function, as measured using the mini-mental state examination, was significantly improved (*P* < 0.05) (61), and the recurrence rate of headache was significantly lower than in the CT group (*P* < 0.05 in all cases) (50, 56). Two studies showed that clinical symptom relief time was significantly shorter in the combination group (*P* < 0.05 in all cases) (52, 73).

#### Safety

**Figure 6:**
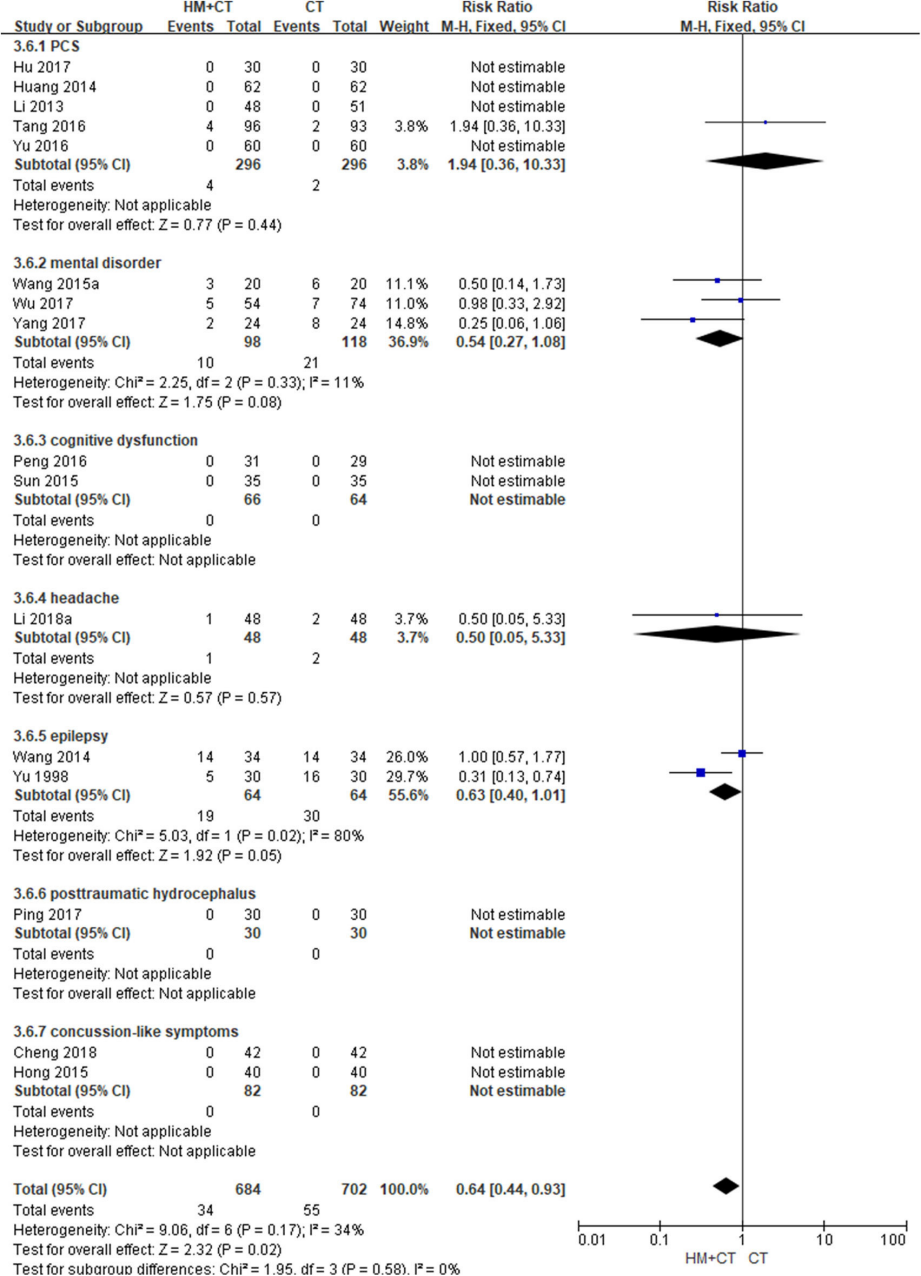
Adverse event (Comparison of herbal medicine combined with conventional treatment vs. conventional treatment alone).

Sixteen studies (51, 55, 56, 61–64, 66, 70, 71, 73, 76, 77, 79, 80, 82) reported the incidence of AEs during the treatment period. The meta-analysis showed that the incidence of AEs was significantly lower in the HM plus CT group than in the CT alone group (RR: 0.64, 95% CI: 0.44–0.93, and *I*^2^ = 34%). Three studies (62, 64, 66) reported TESS scores after treatment in patients with mental disorder. The results showed that TESS scores were significantly lower in the combination group than in the CT group (MD: −1.05, 95% CI: −1.46 to −0.64, and *I*^2^ = 85%; Table 2; Figure 6) (Supplemental Digital Content 3).

### Quality of Evidence

In the studies that compared HM with CT, the quality of evidence was graded as “very low” or “low” (Table 2). Additionally, the quality of evidence was graded as “very low” to “moderate” in studies that compared HM with a placebo, as well as in those that compared HM plus CT with CT alone (Table 2). The main reason for these low grades was the high risk of bias of the included RCTs. Furthermore, most findings had low precision because they did not fulfill the optimal sample size and had wide CIs. Indirect outcome measures also lowered the quality of evidence, especially in studies that measured TER as an outcome.

### Publication Bias

**Figure 7:**
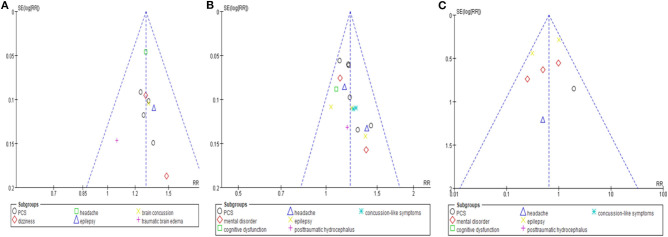
Funnel plots of the meta-analysis. **(A)** Total effective rate based on clinical symptom. Comparison: herbal medicine vs. conventional treatment. **(B)** Total effective rate based on clinical symptom. Comparison: herbal medicine combined with conventional treatment vs. conventional treatment. **(C)** Adverse events. Comparison: herbal medicine combined with conventional treatment vs. conventional treatment.

No evidence of publication bias emerged from the funnel plots of TER based on clinical symptoms in studies that compared the effectiveness of HM with that of CT, or in studies that compared the effectiveness of HM plus CT with that of CT alone. Furthermore, the funnel plot comparing AE incidence between the HM plus CT group and the CT alone group was also symmetrical (Figure 7).

## Discussion

This review aimed to assess the effectiveness and safety of HM as a monotherapy or adjunctive therapy to conventional treatment for TBI. We conducted a comprehensive and systematic search of English, Korean, Chinese, and Japanese-language databases and retrieved a total of 37 RCTs (46–82).

In summary, when comparing HM with CT, there was no conclusive evidence in functional outcome or consciousness state in patients with traumatic brain edema because there was only one study. However, the function measured by Fugl–Meyer assessment, BI, and NIHSS was significantly improved when HM was added to CT in studies that focused on symptomatic treatment or rehabilitation. Results regarding QoL were inconsistent between the two groups after treatment. The present meta-analysis showed that the TER of various symptoms showed significantly better results in the HM group in all comparisons. However, TER is a non-validated outcome measure that is secondarily processed, and thus, assertions regarding HM's effectiveness cannot be made confidently. Regarding the safety of HM, none of the study participants showed obvious abnormalities in electrocardiogram examinations or laboratory tests, such as the blood routine, urine routine, fecal routine, and liver and kidney function tests. There was no difference in the incidence of AEs between the two groups when HM monotherapy was compared with CT or placebo. Conversely, the incidence of AEs and TESS was significantly better in the HM plus CT group than in the CT alone group. However, the risk of bias in the included studies was generally high, whereas the quality of evidence of the main findings was generally low; thus, only limited confidence can be placed in the estimate of the effect, that is, the true effect may be different from the estimate.

Interestingly, pattern identification based on blood stasis was most frequently used in the included studies. In addition, the most commonly used HM was *Xuefuzhuyu* decoction, and the commonly used single herbs comprising the HM were *Cnidii Rhizoma, Angelicae Gigantis Radix, Persicae Semen, Carthami Flos*, and *Paeoniae Radix Rubra*, which improve blood stasis (84, 85). In East-Asian traditional medicine, blood stasis is considered the main pathology in traumatic injury (84). According to this pathological concept, blood stasis-removing therapy is widely used to treat TBI in clinical practice, and some clinical evidence has shown that blood stasis-removing HM is effective in the treatment of TBI (86, 87). Our review does not prove that blood stasis-removing HM is effective in improving TBI, but suggests that this type of herbal medicine is promising in the field of research for TBI treatment in the future.

Many studies have tried to explain the mechanism through which HM functions in TBI, showing that HM decreases neuronal injury by increasing superoxide dismutase and catalase activities, as well as by suppressing the expression of interleukin (IL)-1, IL-6, nuclear factor kappa B, and glial fibrillary acidic protein (88). Another study showed that HM protected a rat model of TBI, possibly via immune-promoting, anti-inflammatory, and neuroprotective effects (89). However, the underlying mechanism of HM in the treatment of TBI is still not fully understood; future studies should address this question to help establish an optimal management strategy for BI.

Our review had the following limitations. Firstly, although we conducted a systematic and comprehensive search in English, Korean, Chinese, and Japanese databases, most studies were conducted and published in China. This may have resulted in reporting biases, such as language and location bias. In addition, many studies assessed TER, which is a secondarily processed outcome measure according to certain criteria, and the meta-analysis showed significant results suggesting better outcomes in the HM group. However, this non-standardized outcome measure may have caused outcome reporting bias, and the results may not have been reliable. Secondly, most of the included studies were not of high quality. In particular, many had a high risk of performance bias. Therefore, our confidence in the effect estimate, as assessed using GRADE methodology, was low. Thirdly, we attempted to perform subgroup analysis in terms of either the objective of intervention (acute management or rehabilitation) or the TBI severity, as described in the study protocol (30). However, few studies clearly specified the objective of intervention or the severity of TBI in a subgroup analysis. Finally, although we performed subgroup analysis according to different target symptoms of TBI to address heterogeneity, we could not resolve clinical heterogeneity because the participants had diverse clinical characteristics and a wide range of interventions were used in the included studies. Relatedly, because the studies showed clinical heterogeneity, we performed only a few quantitative syntheses.

The following recommendations may be considered in future studies. To evaluate the effectiveness of HM in PCS, participants should be enrolled using standardized diagnostic criteria, such as the international statistical classification of diseases and related health problems or the diagnostic and statistical manual of mental disorders. In addition, the multi-compound, multi-target nature of HM may improve a wide range of symptoms after TBI, such as PCS; therefore, the underlying molecular mechanism of HM should be studied. Particularly, priority should be given to HM and/or herb, which are especially known for ameliorating blood stasis, in further HM researches on TBI. To optimize the use of HM during treatment of TBI and to resolve the clinical heterogeneity, future studies should characterize the participants in detail, with particular focus on TBI severity and target symptoms after TBI, such as headache, mental disorder, and cognitive dysfunction, and on the objectives of HM, such as acute management or rehabilitation. In PCS, validated disease specific tools should be adopted to evaluate the effect of HM on various symptoms and deficits; these may include the Rivermead Postconcussion Symptoms Questionnaire, the World Health Organization Disability Assessment Schedule 2.0, and the British Columbia Post-concussion Symptom Inventory-Short Form (90). Finally, only three of the retrieved studies compared HM with a placebo and these showed marked clinical heterogeneity, and thus, we could not draw a definite conclusion about the efficacy of HM. Blinding of participants and personnel using placebo with the same taste, flavor, and formulation should be conducted to avoid performance bias. In future, rigorously conducted, placebo-controlled trials to evaluate the efficacy of HM in TBI should be performed considering the above implications.

## Conclusion

The current evidence suggests that there is insufficient evidence for recommending HM for TBI in clinical practice. Although some RCTs reported that HM as an adjuvant therapy to CT may have benefits for some functional outcomes of TBI, the low quality of evidence significantly limited its reliability. Therefore, further rigorous, well-designed, high quality, placebo-controlled RCTs should be conducted to confirm these results.

## Data Availability Statement

The data used to support the findings of this study are included in the article.

## Author Contributions

This study was conceptualized by JL. BL and C-YK performed the literature search, study selection, data extraction, and quality assessment using the risk of bias tool and GRADE approach. BL analyzed the data and C-YK critically double-checked the data analysis. BL and C-YK drafted the manuscript. All authors interpreted the data and critically reviewed the manuscript. The draft was reviewed and edited by HK, JL, and H-GJ. Resources were provided by JL. This study was supervised by HK and H-GJ. All authors approved the final manuscript.

## Conflict of Interest

JL was employed by the company CY Pharma Co. The remaining authors declare that the research was conducted in the absence of any commercial or financial relationships that could be construed as a potential conflict of interest.

## Abbreviations

AEs: adverse events
AMED: the Allied and Complementary Medicine Database
BI: Barthel index
CDC: the Centers for Disease Control and Prevention
CENTRAL: the Cochrane Central Register of Controlled Trials
CIM: complementary and integrative medicine
CINAHL: the Cumulative Index to Nursing and Allied Health Literature
Cis: confidence intervals
CNKI: China National Knowledge Infrastructure
CT: conventional treatment
GCS: the Glasgow Coma Scale
GOS: Glasgow outcome scale
HM: herbal medicine
IRB: institutional review board
KCI: Korea Citation Index
KISS: Korean studies Information Service System
KMbase: Korean Medical Database
MD: mean difference
NIHSS: the National Institute Of Health Stroke Scale
OASIS: Oriental Medicine Advanced Searching Integrated System
PCS: post-concussion syndrome
PRISMA: the Preferred Reporting Items for Systematic Reviews and Meta-Analyses
QoL: quality of life
RCTs: randomized controlled trials
RISS: Research Information Service System
RR: risk ratio
SF-36: the 36-Item Short Form Health Survey
TBI: traumatic brain injury
TER: total effective rate
TESS: the treatment emergent symptom scale.
