# Supplementary material for: Herbal Medicine for Traumatic Brain Injury: A Systematic Review and Meta-Analysis of Randomized Controlled Trials and Limitations
Source: Front Neurol. 2020 Sep 18;11:772. doi: 10.3389/fneur.2020.00772 (PMC7530840; doi:10.3389/fneur.2020.00772)
Supplement: Supplementary file 1 [file Data_Sheet_1.docx]

**Supplemental Digital Content**

Supplemental Digital Content 1. Search terms used in each database

Supplemental Digital Content 2. Details of herbal medicine used in the included studies

Supplemental Digital Content 3. Forest plots of the meta-analysis

Supplemental Digital Content 4. Results of sensitivity analysis after removal of studies with a high risk of bias

Supplemental Digital Content 5. PRISMA 2009 checklist

Supplemental Digital Content 1. Search terms used in each database

**Medline via PubMed**

|  | Searches | Results (2018/12/2) | Results (2019/7/27) |
| --- | --- | --- | --- |
| #1 | “Brain Injuries, Traumatic”[MH] OR “traumatic brain injury”[TIAB] OR TBI[TIAB] OR “head injury”[TIAB] OR “head trauma”[TIAB] OR “brain injuries”[MH] OR “brain injury”[TIAB] OR “brain trauma”[TIAB] OR “cerebral injury”[TIAB] OR “cerebral trauma”[TIAB] OR “concussion”[TIAB] OR “brain concussion”[MH] OR “brain concussion”[TIAB] OR “cerebral concussion”[TIAB] OR “craniocerebral trauma”[MH] OR “craniocerebral trauma”[TIAB] OR “cerebrovascular trauma”[MH] OR “cerebrovascular trauma”[TIAB] OR "Coma, Post-Head Injury"[MH] OR “diffuse axonal injury”[MH] OR “diffuse axonal injury”[TIAB] OR “traumatic axonal injury”[TIAB] OR “post-concussion syndrome”[MH] OR “post concussion syndrome”[TIAB] | 196156 | 198455 |
| #2 | “Plants, Medicinal”[MH] OR “Drugs, Chinese Herbal”[MH] OR “Medicine, Chinese Traditional”[MH] OR “Medicine, Kampo”[MH] OR “Medicine, Korean Traditional”[MH] OR “Herbal Medicine”[MH] OR “Prescription Drugs”[MH] OR “traditional Korean medicine”[TIAB] OR “traditional Chinese medicine”[TIAB] OR “traditional oriental medicine”[TIAB] OR “Kampo medicine”[TIAB] OR “alternative medicine”[TIAB] OR “complementary medicine”[TIAB] OR herb[TIAB] OR decoction[TIAB] OR botanic[TIAB] | 137106 | 142147 |
| #3 | #1 AND #2 | **410** | **440** |

**EMBASE via Elsevier**

|  | Searches | Results (2018/12/2) | Results (2019/7/27) |
| --- | --- | --- | --- |
| #1 | ‘traumatic brain injury’/exp OR ‘traumatic brain injury’ OR TBI OR ‘head injury’/exp OR ‘head injury’ OR ‘head trauma’ OR ‘brain injury’/exp OR ‘brain injury’ OR ‘brain trauma’ OR ‘cerebral injury’ OR ‘cerebral trauma’ OR ‘brain concussion’/exp OR ‘brain concussion’ OR ‘cerebral concussion’ OR ‘craniocerebral trauma’ OR ‘cerebrovascular trauma’ OR ‘post-head injury’ OR ‘diffuse axonal injury’/exp OR ‘diffuse axonal injury’ OR ‘traumatic axonal injury’ OR ‘postconcussion syndrome’/exp OR ‘postconcussion syndrome’ OR ‘post-concussion syndrome’ | 318574 | 330467 |
| #2 | ‘medicinal plant’/exp OR ‘medicinal plant’ OR ‘herbaceous agent’/exp OR ‘herbaceous agent’ OR ‘chinese medicine’/exp OR ‘chinese medicine’ OR ‘kampo medicine’/exp OR ‘kampo medicine’ OR ‘kampo medicine (drug)’/exp OR ‘kampo medicine (drug)’ OR ‘korean medicine’/exp OR ‘korean medicine’ OR ‘herbal medicine’/exp OR ‘herbal medicine’ OR ‘prescription drug’/exp OR ‘prescription drug’ OR ‘oriental medicine’/exp OR ‘oriental medicine’ OR ‘alternative medicine’/exp OR ‘alternative medicine’ OR ‘complementary medicine’ OR ‘herb’/exp OR ‘herb’ OR ‘decoction’ OR ‘botanic’ | 448088 | 478523 |
| #3 | #1 AND #2 | **2334** | **2528** |

**CENTRAL**

|  | Searches | Results (2018/12/2) | Results (2019/7/27) |
| --- | --- | --- | --- |
| #1 | MeSH descriptor: [Brain Injuries, Traumatic] explode all trees | 443 | 514 |
| #2 | MeSH descriptor: [brain injuries] explode all trees | 1867 | 1960 |
| #3 | MeSH descriptor: [brain concussion] explode all trees | 248 | 272 |
| #4 | MeSH descriptor: [craniocerebral trauma] explode all trees | 2986 | 3103 |
| #5 | MeSH descriptor: [cerebrovascular trauma] explode all trees | 30 | 32 |
| #6 | MeSH descriptor: [Coma, Post-Head Injury] explode all trees | 5 | 5 |
| #7 | MeSH descriptor: [diffuse axonal injury] explode all trees | 10 | 10 |
| #8 | MeSH descriptor: [post-concussion syndrome] explode all trees | 96 | 107 |
| #9 | (“traumatic brain injury” OR TBI OR “head injury” OR “head trauma” OR “brain injury” OR “brain trauma” OR “cerebral injury” OR “cerebral trauma” OR “concussion” OR “brain concussion” OR “cerebral concussion” OR “craniocerebral trauma” OR “cerebrovascular trauma” OR “diffuse axonal injury” OR “traumatic axonal injury” OR “post concussion syndrome”):ti,ab,kw | 5813 | 7365 |
| #10 | #1 OR #2 OR #3 OR #4 OR #5 OR #6 OR #7 OR #8 OR #9 | 6855 | 8431 |
| #11 | MeSH descriptor: [Plants, Medicinal] explode all trees | 933 | 934 |
| #12 | MeSH descriptor: [Drugs, Chinese Herbal] explode all trees | 3295 | 3400 |
| #13 | MeSH descriptor: [Medicine, Chinese Traditional] explode all trees | 1043 | 1082 |
| #14 | MeSH descriptor: [Medicine, Kampo] explode all trees | 38 | 40 |
| #15 | MeSH descriptor: [Medicine, Korean Traditional] explode all trees | 24 | 26 |
| #16 | MeSH descriptor: [Herbal Medicine] explode all trees | 49 | 54 |
| #17 | MeSH descriptor: [Prescription Drugs] explode all trees | 110 | 110 |
| #18 | (“traditional Korean medicine” OR “traditional Chinese medicine” OR “Traditional oriental medicine” OR “Kampo medicine” OR “alternative medicine” OR “complementary medicine” OR herb OR decoction OR botanic):ti,ab,kw | 7569 | 10465 |
| #19 | #11 OR #12 OR #13 OR #14 OR #15 OR #16 OR #17 OR #18 | 11329 | 14309 |
| #20 | (#10 AND #19) in Trials | **34** | **51** |

**AMED via EBSCO**

|  | Searches | Results (2018/12/2) | Results (2019/7/27) |
| --- | --- | --- | --- |
| #1 | “Brain Injuries, Traumatic”[SU] OR “traumatic brain injury”[TX] OR TBI[TX] OR “head injury”[TX] OR “head trauma”[TX] OR “brain injuries”[SU] OR “brain injury”[TX] OR “brain trauma”[TX] OR “cerebral injury”[TX] OR “cerebral trauma”[TX] OR “concussion”[TX] OR “brain concussion”[SU] OR “brain concussion”[TX] OR “cerebral concussion”[TX] OR “craniocerebral trauma”[SU] OR “craniocerebral trauma”[TX] OR “cerebrovascular trauma”[SU] OR “cerebrovascular trauma”[TX] OR "Coma, Post-Head Injury"[SU] OR “diffuse axonal injury”[SU] OR “diffuse axonal injury”[TX] OR “traumatic axonal injury”[TX] OR “post-concussion syndrome”[SU] OR “post concussion syndrome”[TX] | 5678 | 6140 |
| #2 | “Plants, Medicinal”[SU] OR “Drugs, Chinese Herbal”[SU] OR “Medicine, Chinese Traditional”[SU] OR “Medicine, Kampo”[SU] OR “Medicine, Korean Traditional”[SU] OR “Herbal Medicine”[SU] OR “Prescription Drugs”[SU] OR “traditional Korean medicine”[TX] OR “traditional Chinese medicine”[TX] OR “traditional oriental medicine”[TX] OR “Kampo medicine”[TX] OR “alternative medicine”[TX] OR “complementary medicine”[TX] OR herb[TX] OR decoction[TX] OR botanic[TX] | 31176 | 31997 |
| #3 | #1 AND #2 | **46** | **53** |

**CINAHL via EBSCO**

|  | Searches | Results (2018/12/2) | Results (2019/7/27) |
| --- | --- | --- | --- |
| #1 | “Brain Injuries, Traumatic”[MH] OR “traumatic brain injury”[TX] OR TBI[TX] OR “head injury”[TX] OR “head trauma”[TX] OR “brain injuries”[MH] OR “brain injury”[TX] OR “brain trauma”[TX] OR “cerebral injury”[TX] OR “cerebral trauma”[TX] OR “concussion”[TX] OR “brain concussion”[MH] OR “brain concussion”[TX] OR “cerebral concussion”[TX] OR “craniocerebral trauma”[MH] OR “craniocerebral trauma”[TX] OR “cerebrovascular trauma”[MH] OR “cerebrovascular trauma”[TX] OR "Coma, Post-Head Injury"[MH] OR “diffuse axonal injury”[MH] OR “diffuse axonal injury”[TX] OR “traumatic axonal injury”[TX] OR “post-concussion syndrome”[MH] OR “post concussion syndrome”[TX] | 64019 | 69726 |
| #2 | “Plants, Medicinal”[MH] OR “Drugs, Chinese Herbal”[MH] OR “Medicine, Chinese Traditional”[MH] OR “Medicine, Kampo”[MH] OR “Medicine, Korean Traditional”[MH] OR “Herbal Medicine”[MH] OR “Prescription Drugs”[MH] OR “traditional Korean medicine”[TX] OR “traditional Chinese medicine”[TX] OR “traditional oriental medicine”[TX] OR “Kampo medicine”[TX] OR “alternative medicine”[TX] OR “complementary medicine”[TX] OR herb[TX] OR decoction[TX] OR botanic[TX] | 77435 | 81258 |
| #3 | #1 AND #2 | **1291** | **1403** |

**OASIS**

|  | Searches | Results (2018/12/2) | Results (2019/7/27) |
| --- | --- | --- | --- |
| #1 | 외상 AND 뇌 | 14 | 15 |
| #2 | 뇌진탕 | 2 | 2 |
| #3 | 두부외상 | 0 | 0 |
| #4 | #1 OR #2 OR #3 | **16** | **17** |

**KISS**

|  | Searches | Results (2018/12/2) | Results (2019/7/27) |
| --- | --- | --- | --- |
| #1 | (외상 AND 뇌) AND 한약 | 1 | 1 |
| #2 | (뇌진탕 OR 두부외상) AND 한약 | 0 | 0 |
| #3 | #1 OR #2 | **1** | **1** |

**RISS**

|  | Searches | Results (2018/12/2) | Results (2019/7/27) |
| --- | --- | --- | --- |
| #1 | (외상 AND 뇌) AND 한약 | 5 | 5 |
| #2 | (뇌진탕 OR 두부외상) AND 한약 | 0 | 0 |
| #3 | #1 OR #2 | **5** | **5** |

**KMbase**

|  | Searches | Results (2018/12/2) | Results (2019/7/27) |
| --- | --- | --- | --- |
| #1 | (외상 AND 뇌) AND 한약 | 0 | 0 |
| #2 | (뇌진탕 OR 두부외상) AND 한약 | 0 | 0 |
| #3 | #1 OR #2 | **0** | **0** |

**KCI**

|  | Searches | Results (2018/12/2) | Results (2019/7/27) |
| --- | --- | --- | --- |
| #1 | (외상 AND 뇌) AND 한약 | 0 | 0 |
| #2 | (뇌진탕 OR 두부외상) AND 한약 | 0 | 0 |
| #3 | #1 OR #2 | **0** | **0** |

**CNKI**

|  | Searches | Results (2018/12/2) | Results (2019/7/27) |
| --- | --- | --- | --- |
| #1 | (SU='创伤性脑损伤'+'创伤性颅脑损伤'+'脑外伤'+'脑创伤'+'外伤性脑损伤'+'弥漫性轴索损伤'+'创伤性轴索损伤'+'脑震荡'+'**头部外伤**') AND (SU='中药'+'中医药'+'本草'+'汤'+'丸'+'散'+'方'+'颗粒'+'胶囊'+'自拟') | **685** | **699** |

**Wanfang data**

|  | Searches | Results (2018/12/2) | Results (2019/7/27) |
| --- | --- | --- | --- |
| #1 | (主题:(“创伤性脑损伤” + “创伤性颅脑损伤” + “脑外伤” + “脑创伤” + “外伤性脑损伤” + “弥漫性轴索损伤” + “创伤性轴索损伤” + “脑震荡” + “头部外伤”) * 主题:(“中药” + “中医药” + “本草” + “汤” + “丸” + “散” + “方” + “颗粒” + “胶囊” + “自拟”)) | **18671** | **21311** |

**VIP**

|  | Searches | Results (2018/12/2) | Results (2019/7/27) |
| --- | --- | --- | --- |
| #1 | (M=(创伤性脑损伤 OR 创伤性颅脑损伤 OR 脑外伤 OR 脑创伤 OR 外伤性脑损伤 OR 弥漫性轴索损伤 OR 弥漫性轴索损伤 OR 脑震荡 OR 头部外伤)) AND (M=(中药 OR 中医药 OR 本草 OR 汤 OR 丸 OR 散 OR 方 OR 颗粒 OR 胶囊 OR 自拟)) | **580** | **591** |

**CiNii**

|  | Searches | Results (2018/12/2) | Results (2019/7/27) |
| --- | --- | --- | --- |
| #1 | (外傷性脳損傷 OR 脳震盪 OR 頭部外傷 OR 脳外傷 OR ポストヘッド傷害 OR びまん性軸索損傷 OR 外傷性軸索損傷) AND (“traditional Korean medicine” OR “traditional Chinese medicine” OR “Traditional oriental medicine” OR “Kampo medicine” OR “alternative medicine” OR “complementary medicine” OR herb OR decoction OR botanic OR 漢方薬 OR ハーブ OR 散 OR 汤 OR 丸) | **153** | **159** |

Supplemental Digital Content 2. Details of herbal medicine used in the included studies

| **Study ID** | **Name** | **Dosage form** | **Frequency** | **Composition and dosage (per one day)** | **Modifying components** |
| --- | --- | --- | --- | --- | --- |
| Cai 2012 | Angong Niuhuang pill | pill | Once a day | Bovis Calculus, Bubali Cornu, Moschus, Margarita, Cinnabaris, Realgar, Coptidis Rhizoma, Scutellariae Radix, Gardeniae Fructus, Curcumae Radix, Bomeolum | None |
| Cheng 2018 | Yangxue Qingnao granule | granule | Three times a day | Cnidii Rhizoma, Paeoniae Radix, Angelicae Gigantis Radix, Uncariae Ramulus cum Uncus, Rehmanniae Radix Preparata, Spatholobi Caulis, Prunellae Spica, Margaritifera Usta Concha, Cassiae Semen, Asiasari Radix Et Rhizoma, Corydalis Tuber | None |
| Ding 2007 | Huoxue Xingnao decoction | decoction | Twice a day | Astragali Radix 30 g, Salviae Miltiorrhizae Radix 15 g, Notoginseng Radix Et Rhizoma 10 g, Spatholobi Caulis 20 g, Curcumae Radix 10 g, Acori Graminei Rhizoma 10 g, Polygalae Radix 10 g, Cnidii Rhizoma 10 g, Glycyrrhizae Radix Et Rhizoma | -headche: Angelicae Dahuricae Radix, Uncariae Ramulus cum Uncus -dizziness, nausea, vomiting: Pinelliae Tuber, Atractylodis Rhizoma Alba, Gastrodiae Rhizoma -agitation, insomnia: Zizyphi Semen, Polygoni Multiflori Caulis |
| Gao 2016 | Wendan decoction plus Banxia Baizhu Tianma decoction | decoction | Once a day | Pinelliae Tuber 12 g, Atractylodis Rhizoma Alba 10 g, Gastrodiae Rhizoma 18 g, Poria Sclerotium 10 g, Citri Unshius Pericarpium 12 g, Phyllostachyos Caulis in Taeniam 18 g, Coptidis Rhizoma 9 g, Scutellariae Radix 10 g, Acori Graminei Rhizoma 12 g | None |
| Gong 2019 | Xuefuzhuyu decoction | decoction | Twice a day | Persicae Semen 12 g, Carthami Flos 9 g, Achyranthis Radix 9 g, Angelicae Gigantis Radix 9 g, Rehmanniae Radix Recens 9 g, Paeoniae Radix Rubra 6 g, Aurantii Fructus Immaturus 6 g, Platycodonis Radix 5 g, Cnidii Rhizoma 5 g, Bupleuri Radix 3 g, Glycyrrhizae Radix Et Rhizoma 3 g | None |
| Gu 2016 | Jieyu Tongqiao decoction | granule | Twice a day | Angelicae Gigantis Radix 15 g, Cnidii Rhizoma 12 g, Salviae Miltiorrhizae Radix 18 g, Paeoniae Radix Rubra 12 g, Paeoniae Radix 12 g, Bupleuri Radix 10 g, Curcumae Radix 12 g, Acori Graminei Rhizoma 15 g | -qi deficiency with blood stasis: Astragali Radix 30 g, Atractylodis Rhizoma Alba 15 g, Poria Sclerotium 12 g -ascendant hyperactivity of liver yang: Gastrodiae Rhizoma 10 g, Uncariae Ramulus cum Uncus 12 g, Nardotidis seu Sulculii Concha 15 g, Gentianae Scabrae Radix Et Rhizoma 6 g -kidney deficiency: Morindae Radix 12 g, Eucommiae Cortex 12 g, Alpiniae Oxyphyllae Fructus 12 g -deficiency of qi and blood: Astragali Radix 18 g, Codonopsis Pilosulae Radix 15 g, Atractylodis Rhizoma Alba 12 g, Dioscoreae Rhizoma 12 g |
| Hong 2015 | Yangxue Qingnao granule | granule | Three times a day | Angelicae Gigantis Radix, Cnidii Rhizoma, Paeoniae Radix, Rehmanniae Radix Preparata | None |
| Hu 2017 | Xiaoyaosan | decoction | Twice a day | Angelicae Gigantis Radix 15 g, Bupleuri Radix 15 g, Paeoniae Radix 15 g, Poria Sclerotium 10 g, Atractylodis Rhizoma Alba 10 g, Menthae Herba 10 g, Zingiberis Rhizoma Recens 10 g, Glycyrrhizae Radix Et Rhizoma 6 g | -obvious liver qi stagnation: Curcumae Radix, Cyperi Rhizoma -obvious blood stasis due to qi stagnation: Cnidii Rhizoma, Persicae Semen, Carthami Flos -obvious depressed liver qi transforming into fire: Gardeniae Fructus, Moutan Radicis Cortex -obvious ascendant hyperactivity of liver yang: Ostreae Testa, Uncariae Ramulus cum Uncus -obvious liver-kidney yin deficiency: Achyranthis Radix, Rehmanniae Radix Preparata -obvious liver blood deficiency: Lycii Fructus, Asini Corii Colla -obvious liver depression and spleen deficiency: Codonopsis Pilosulae Radix, Astragali Radix |
| Huang 2008 | Decanxiangren decoction | decoction | NR | Rehmanniae Radix Recens 10 g, Codonopsis Pilosulae Radix 10 g, Salviae Miltiorrhizae Radix 10 g, Cyperi Rhizoma 10 g, Aucklandiae Radix 10 g, Cnidii Rhizoma 10 g, Zizyphi Semen 10 g, Thujae Semen 10 g, Schisandrae Fructus 10 g, Poria Sclerotium 10 g, Platycodonis Radix 5 g, Polygalae Radix 10 g, Bupleuri Radix 10 g, Anemarrhenae Rhizoma 10 g, Moutan Radicis Cortex 10 g, Angelicae Gigantis Radix 5 g, Polygoni Multiflori Caulis 10 g | -forehead pain: Angelicae Dahuricae Radix 10 g -occipital pain: Osterici seu Notopterygii Radix Et Rhizoma 10 g -bilateral temple pain: Cnidii Rhizoma 10 g, Schizonepetae Spica 10 g -eyebrow bone pain: Ligustici Tenuissimi Rhizoma Et Radix 10 g -the top of the head pain: Asiasari Radix Et Rhizoma 3 g, Evodiae Fructus 10 g -nape and neck rigidity and pain: Puerariae Radix 30 g, Uncariae Ramulus cum Uncus 10 g -dizziness: Gastrodiae Rhizoma 15 g, Uncariae Ramulus cum Uncus 15 g, Chrysanthmi Flos 15 g -insomnia: Albizia Julibrissin 10 g -vomiting: Inulae Flos 15 g, Phyllostachyos Caulis in Taeniam 10 g, Agastachis Herba 10 g -tinnitus: Magenetitum 30 g, Cicadidae Periostracum 5 g -bitter taste in the mouth and vexation: Scutellariae Radix 10 g, Gardeniae Fructus 10 g -phelgm worsening: Arisaematis Rhizoma 10 g, Poria Sclerotium 10 g -liver qi stagnation: Paeoniae Radix 15 g, Aurantii Fructus Immaturus 10 g -kidney deficiency: Ligustri Fructus 10 g, Polygoni Multiflori Radix 10 g -qi deficiency and lack of strength: Astragali Radix 30 g -hemiplegia or numbness: Spatholobi Caulis 30 g, Trachelospermi Caulis 15 g, Scolopendra 2-3 pieces |
| Huang 2014 | Yangxue Qingnao granule | granule | Three times a day | Angelicae Gigantis Radix, Cnidii Rhizoma, Rehmanniae Radix Preparata, Margaritifera Usta Concha, Cassiae Semen, Prunellae Spica, Paeoniae Radix | None |
| Lan 2019 | modified Taohong Siwu decoction | granule | Three times a day | Persicae Semen 3 g, Carthami Flos 3 g, Angelicae Gigantis Radix 5 g, Rehmanniae Radix Preparata 5 g, Cnidii Rhizoma 3 g, Paeoniae Radix Rubra 3 g, Notoginseng Radix Et Rhizoma 3 g, Rubiae Radix 3 g | -according to injured part: Acori Graminei Rhizoma 3 g, Curcumae Radix 3 g, Ligustici Tenuissimi Rhizoma Et Radix 3 g or Bupleuri Radix 3 g, Platycodonis Radix 3 g, Aurantii Fructus Immaturus 3 g -according to degree of swelling and pain: Olibanum 3 g, Myrrha 3 g, Corydalis Tuber 3 g |
| Li 2013 | self-made Chinese herbal medicine | decoction | NR | Salviae Miltiorrhizae Radix 20 g, Angelicae Gigantis Radix 15 g, Cnidii Rhizoma 12 g, Bupleuri Radix 12 g, Zingiberis Rhizoma Recens 2 pieces, Persicae Semen 10 g, Carthami Flos 10 g, Paeoniae Radix Rubra 10 g, Angelicae Dahuricae Radix 10 g, Aurantii Fructus Immaturus 10 g, Cyperi Rhizoma 10 g, Citri Unshius Pericarpium 6 g | -dizziness, tinnitus: Nardotidis seu Sulculii Concha 15 g, Uncariae Ramulus cum Uncus 12 g, Gastrodiae Rhizoma 10 g |
| Li 2018a | Chuanxiong Qingnao granule | granule | Three times a day | Cnidii Rhizoma, Angelicae Gigantis Radix, Saposhnikoviae Radix, Angelicae Dahuricae Radix, Liriopis seu Ophiopogonis Tuber, Asiasari Radix Et Rhizoma, Osterici seu Notopterygii Radix Et Rhizoma, Araliae Continentalis Radix, Atractylodis Rhizoma, Chrysanthmi Flos, Viticis Fructus, Scutellariae Radix, Glycyrrhizae Radix Et Rhizoma | None |
| Li 2018b | Buyang Huanwu decoction | decoction | Twice a day | Astragali Radix 120 g, Angelicae Gigantis Radix 6 g, Paeoniae Radix Rubra 5 g, Lumbricus 3 g, Cnidii Rhizoma 3 g, Carthami Flos 3 g, Persicae Semen 3 g | None |
| Liu 2005 | Canqiwuweizi pill | pill | Three times a day | Schisandrae Fructus, Codonopsis Pilosulae Radix, Astragali Radix, Zizyphi Semen | None |
| Liu 2008 | Tongqiao Pingyu pill | pill | Three times a day | Astragali Radix 30 g, Salviae Miltiorrhizae Radix 15 g, Paeoniae Radix Rubra 10 g, Persicae Semen 6 g, Bupleuri Radix 12 g, Aurantii Fructus Immaturus 8 g, Achyranthis Radix 15 g, Platycodonis Radix 9 g, Acori Graminei Rhizoma 10 g, Chalcocitum 20 g, Atractylodis Rhizoma Alba 15 g, Hirudo 6 g | None |
| Ma 2017 | modified Wulingsan | decoction | NR | Polyporus 9 g, Alismatis Rhizoma 15 g, Atractylodis Rhizoma Alba 9 g, Poria Sclerotium 9 g, Cinnamomi Ramulus 6 g, Cnidii Rhizoma 9 g, Achyranthis Radix 6 g, Ophicalcitum 6 g | None |
| Peng 2016 | Xingnao Zhuyu decoction | decoction | Twice a day | Persicae Semen 12 g, Carthami Flos 6 g, Achyranthis Radix 15 g, Angelicae Gigantis Radix 15 g, Cnidii Rhizoma 15 g, Bupleuri Radix 15 g, Polygalae Radix 6 g, Poria Sclerotium 15 g, Acori Graminei Rhizoma 10 g, Salviae Miltiorrhizae Radix 15 g, Gastrodiae Rhizoma 12 g | None |
| Ping 2017 | Banxia Baizhu Tianma decoction | decoction | Three times a day | Pinelliae Tuber 15 g, Atractylodis Rhizoma Alba 30 g, Gastrodiae Rhizoma 10 g, Poria Sclerotium 20 g, Citri Unshius Pericarpium 15 g, Glycyrrhizae Radix Et Rhizoma 15 g | -severe headache: Olibanum 15 g, Myrrha 15 g, Notoginseng Radix Et Rhizoma 3 g -fear of cold and vomiting: Zingiberis Rhizoma 10 g, Massa Medicata Fermentata 15 g, Hordei Fructus Germinatus 30 g -unconsciousness: Acori Graminei Rhizoma 30 g, Carthami Flos 10 g -physical activity disorder: Spatholobi Caulis 30 g, Lumbricus 20 g |
| Qiu 2016 | Buyang Huanwu decoction | decoction | Twice a day | Astragali Radix 30-60 g, Lumbricus 8 g, Persicae Semen 10 g, Angelicae Gigantis Radix 10 g, Carthami Flos 10 g, Polygalae Radix 10 g, Paeoniae Radix 10 g, Poria Sclertum Cum Pini Radix 15 g, Cnidii Rhizoma 15 g, Zizyphi Semen 15 g, Salviae Miltiorrhizae Radix 15 g, Longan Arillus 30 g | -obvious headache: Gastrodiae Rhizoma 15-20 g, Scolopendra 1-2 piece, Cnidii Rhizoma 30 g -hemiplegia: Hirudo 6 g, Zingiberis Rhizoma Recens 6 g, Atractylodis Rhizoma Alba 15 g -insomnia and forgetfulness: Poria Sclertum Cum Pini Radix 20 g, Polygoni Multiflori Caulis 30 g, Albizziae Cortex 15 g |
| Sun 2015 | Huishen Xingnao decoction | decoction | Twice a day | Paeoniae Radix Rubra 10 g, Persicae Semen 10 g, Cnidii Rhizoma 10 g, Acori Graminei Rhizoma 10 g, Carthami Flos 10 g, Uncariae Ramulus cum Uncus 10 g, Pinelliae Tuber 10 g, Angelicae Gigantis Radix 10 g, Angelicae Dahuricae Radix 10 g, Bupleuri Radix 10 g, Dens Draconis 30 g, Moschus 0.1 g | None |
| Tang 2016 | Huishen Xingnao decoction | decoction | Twice a day | Dens Draconis 30 g, Paeoniae Radix Rubra 10 g, Persicae Semen 10 g, Cnidii Rhizoma 10 g, Acori Graminei Rhizoma 10 g, Carthami Flos 10 g, Uncariae Ramulus cum Uncus 10 g, Pinelliae Tuber 10 g, Angelicae Gigantis Radix 10 g, Angelicae Dahuricae Radix 10 g, Bupleuri Radix 10 g, Moschus 0.1 g | None |
| Theadom 2018 | MLC901 (0.8 g capsules) | capsule | Three times a day | Astragali Radix, Salviae Miltiorrhizae Radix, Paeoniae Radix, Cnidii Rhizoma, Angelicae Gigantis Radix, Carthami Flos, Persicae Semen, Polygalae Radix, Acori Graminei Rhizoma (2 caps per time) | None |
| Wang 2009 | Yangxue Qingnao granule | granule | Three times a day | Cnidii Rhizoma, Paeoniae Radix, Angelicae Gigantis Radix, Uncariae Ramulus cum Uncus, Rehmanniae Radix Preparata, Spatholobi Caulis, Prunellae Spica, Margaritifera Usta Concha, Cassiae Semen, Asiasari Radix Et Rhizoma, Corydalis Tuber | None |
| Wang 2013 | Naoxintong capsule | capsule | Three times a day | Astragali Radix, Paeoniae Radix Rubra, Salviae Miltiorrhizae Radix, Angelicae Gigantis Radix, Cnidii Rhizoma, Persicae Semen, Carthami Flos, Olibanum Myrrha, Spatholobi Caulis, Achyranthis Radix, Cinnamomi Ramulus, Mori Ramulus, Lumbricus, Scorpio, Hirudo (2 caps per time) | None |
| Wang 2014 | NR | decoction | Once a day | Curcumae Radix 120 g, Massa Medicata Fermentata 120 g, Atractylodis Rhizoma Alba 90 g, Arisaematis Rhizoma 90 g, Poria Sclertum Cum Pini Radix 90 g, Acori Graminei Rhizoma 90 g, Lumbricus 60 g, Batryticatus Bombyx 60 g, Alumen 60 g, Gastrodiae Rhizoma 60 g, Scorpio 30 g, Ponciri Fructus Immaturus 30 g, Cinnabaris 20 g | None |
| Wang 2015a | Xuefuzhuyu decoction | decoction | Twice a day | Persicae Semen 15 g, Carthami Flos 5 g, Angelicae Gigantis Radix 10 g, Cnidii Rhizoma 10 g, Paeoniae Radix Rubra 20 g, Bupleuri Radix 15 g, Paeoniae Radix 30 g, Platycodonis Radix 10 g, Curcumae Radix 15 g, Aurantii Fructus Immaturus 10 g, Achyranthis Radix 15 g, Rehmanniae Radix Recens 15 g, Liriopis seu Ophiopogonis Tuber 20 g, Albizziae Cortex 8 g, Zizyphi Semen 15 g, Polygalae Radix 8 g, Acori Graminei Rhizoma 15 g, Citri Unshius Pericarpium 8 g, Glycyrrhizae Radix Et Rhizoma | -profuse phlegm: Pinelliae Tuber 10 g, Concha Arcae 15 g, Arisaematis Rhizoma 10 g, Poria Sclerotium 30 g -poor sleep: Margaritifera Usta Concha 30 g, Ostreae Testa 30 g -nausea and vomiting: Magnoliae Cortex 15 g, Bambusae Sulcus 15 g |
| Wang 2015b | Xuefuzhuyu decoction | decoction | Twice a day | Fossilia Ossis Mastodi 20 g, Uncariae Ramulus cum Uncus 15 g, Persicae Semen 12 g, Gastrodiae Rhizoma 10 g, Arisaematis Rhizoma 10 g, Angelicae Gigantis Radix 9 g, Achyranthis Radix 9 g, Rehmanniae Radix Recens 9 g, Carthami Flos 9 g, Aurantii Fructus Immaturus 6 g, Paeoniae Radix Rubra 6 g, Bupleuri Radix 5 g, Cnidii Rhizoma 5 g, Platycodonis Radix 5 g, Scorpio 3 g, Glycyrrhizae Radix Et Rhizoma 3 g | -heavier blood stasis: Sparganii Rhizoma 5 g, Curcumae Rhizoma 5 g -head trauma: Salviae Miltiorrhizae Radix 5 g, Notoginseng Radix Et Rhizoma 5 g -liver-kidney yin deficiency: remove Persicae Semen, Carthami Flos, add Polygoni Multiflori Radix 5 g, Rehmanniae Radix Preparata 5 g, Lycii Fructus 5 g -dual deficiency of the heart and spleen: remove Persicae Semen, Carthami Flos, add Codonopsis Pilosulae Radix 5 g, Atractylodis Rhizoma Alba 5 g, Poria Sclerotium 5 g |
| Wu 2012 | Tongqiao huoxue decoction | decoction | Twice a day | Cnidii Rhizoma 10 g, Paeoniae Radix Rubra 15 g, Persicae Semen 6 g, Carthami Flos 5 g, Zingiberis Rhizoma Recens 9 g, Pinelliae Tuber 9 g, Angelicae Dahuricae Radix 6 g, Acori Graminei Rhizoma 6 g, Zizyphi Fructus 6 things, Alismatis Rhizoma 10 g, Aurantii Fructus Immaturus 10 g | -deficiency of qi and blood: Astragali Radix, Angelicae Gigantis Radix -heavier blood stasis: Scorpio, Scolopendra -fever: Chrysanthmi Flos, Scutellariae Radix -cold: Cinnamomi Ramulus, Asiasari Radix Et Rhizoma -insomnia and forgetfulness: Zizyphi Semen, Thujae Semen, Ostreae Testa |
| Wu 2017 | Xuefuzhuyu decoction | decoction | Twice a day | Paeoniae Radix 30 g, Liriopis seu Ophiopogonis Tuber 20 g, Paeoniae Radix Rubra 20 g, Zizyphi Semen 15 g, Achyranthis Radix 15 g, Rehmanniae Radix Recens 15 g, Acori Graminei Rhizoma 15 g, Bupleuri Radix 15 g, Curcumae Radix 15 g, Persicae Semen 15 g, Aurantii Fructus Immaturus 10 g, Angelicae Gigantis Radix 10 g, Platycodonis Radix 10 g, Cnidii Rhizoma 10 g, Albizziae Cortex 8 g, Polygalae Radix 8 g, Citri Unshius Pericarpium 8 g, Glycyrrhizae Radix Et Rhizoma 6 g, Carthami Flos 5 g | -profuse phlegm: Poria Sclerotium 30 g, Concha Arcae 15 g, Pinelliae Tuber 10 g, Arisaematis Rhizoma 10 g -nausea and vomiting: Magnoliae Cortex 15 g, Bambusae Sulcus 15 g -poor sleep quality: Concha Margaritifera 30 g, Fossilia Ossis Mastodi 30 g, Ostreae Testa 30 g |
| Xu 2012 | Danqin Xiaoyu mixture | decoction | Three times a day | Bupleuri Radix, Angelicae Gigantis Radix, Paeoniae Radix, Poria Sclerotium, Glycyrrhizae Radix Et Rhizoma, Scutellariae Radix, Moutan Radicis Cortex, Menthae Herba, Rehmanniae Radix Recens, Dioscoreae Rhizoma | None |
| Xu 2017 | Huishen Xingnao decoction | decoction | Twice a day | Paeoniae Radix Rubra 10 g, Angelicae Dahuricae Radix 10 g, Angelicae Gigantis Radix 10 g, Persicae Semen 10 g, Bupleuri Radix 10 g, Cnidii Rhizoma 10 g, Acori Graminei Rhizoma 10 g, Pinelliae Tuber 10 g, Carthami Flos 10 g, Uncariae Ramulus cum Uncus 10 g, Moschus 0.1 g, Dens Draconis 30 g | None |
| Xu 2018 | modified Wendan decoction | decoction | NR | Glycyrrhizae Radix Et Rhizoma, Poria Sclerotium, Pinelliae Tuber, Ponciri Fructus Immaturus, Citri Unshius Pericarpium, Phyllostachyos Caulis in Taeniam | -dry stool, anorexia: Forsythiae Fructus, Trichosanthis Caulis -fear of cold and cold limbs, fatigue, depression: Codonopsis Pilosulae Radix, Amomi Fructus Rotundus, Atractylodis Rhizoma Alba -poor qi movement and static blood obstructing the collaterals: Curcumae Radix, Carthami Flos, Persicae Semen, Salviae Miltiorrhizae Radix -disquieted consciousness: Polygalae Radix, Carthami Flos, Persicae Semen, Salviae Miltiorrhizae Radix, Bambusae Concretio Silicea |
| Yang 2017 | Xuefuzhuyu decoction | decoction | Twice a day | Paeoniae Radix Rubra 20 g, Liriopis seu Ophiopogonis Tuber 20 g, Persicae Semen 15 g, Bupleuri Radix 15 g, Curcumae Radix 15 g, Zizyphi Semen 15 g, Achyranthis Radix 15 g, Rehmanniae Radix Recens 15 g, Acori Graminei Rhizoma 15 g, Angelicae Gigantis Radix 10 g, Aurantii Fructus Immaturus 10 g, Platycodonis Radix 10 g, Cnidii Rhizoma 10 g, Citri Unshius Pericarpium 18 g, Polygalae Radix 8 g, Albizziae Cortex 8 g, Carthami Flos 5 g | -profuse phlegm: Poria Sclerotium 30 g, Concha Arcae 15 g, Pinelliae Tuber 10 g, Arisaematis Rhizoma 10 g -nausea and vomiting: Magnoliae Cortex 15 g, Bambusae Sulcus 15 g -poor sleep quality: Concha Margaritifera 30 g, Fossilia Ossis Mastodi 30 g, Ostreae Testa 30 g |
| Yu 1998 | Tongqiao huoxue decoction | decoction | Three times a day | Persicae Semen 10 g, Crocus Sativus 6 g, Zizyphi Fructus 10 g, Cnidii Rhizoma 10 g, Allii Fistulosi Bulbus 3 pieces, Zingiberis Rhizoma Recens 10 g, Angelicae Dahuricae Radix 10 g, Pinelliae Tuber 10 g, Arisaematis Rhizoma 10 g, Citri Unshius Pericarpium 10 g, Poria Sclerotium 10 g, Acori Graminei Rhizoma 10 g, Phyllostachyos Caulis in Taeniam 10 g, Glycyrrhizae Radix Et Rhizoma 6 g, Ginseng Radix 10 g | None |
| Yu 2013 | modified Xuefuzhuyu decoction | decoction | Twice a day | Angelicae Gigantis Radix 9 g, Rehmanniae Radix Recens 9 g, Achyranthis Radix 9 g, Carthami Flos 9 g, Persicae Semen 12 g, Aurantii Fructus Immaturus 6 g, Paeoniae Radix Rubra 6 g, Platycodonis Radix 5 g, Cnidii Rhizoma 5 g, Bupleuri Radix 5 g, Gastrodiae Rhizoma 10 g, Arisaematis Rhizoma 10 g, Uncariae Ramulus cum Uncus 15 g, Fossilia Ossis Mastodi 20 g, Scorpio 3 g, Glycyrrhizae Radix Et Rhizoma 3 g | None |
| Yu 2016 | Xueshuantong capsule | capsule | Three times a day | Notoginseng Radix Et Rhizoma, Astragali Radix, Salviae Miltiorrhizae Radix, Scrophulariae Radix | None |

Abbreviation: NR, not recorded.

Supplemental Digital Content 3. Forest plots of the meta-analysis

1. Comparison of herbal medicine *versus* conventional treatment

1. Glasgow outcome scale


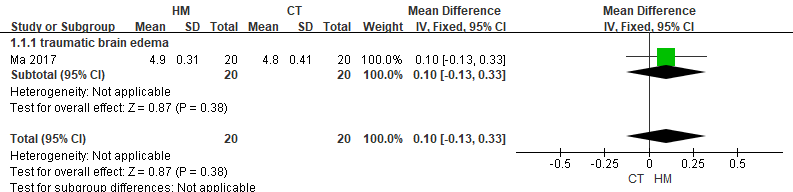


1. Glasgow coma scale


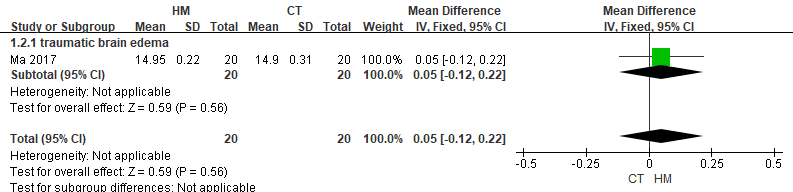


1. Total effective rate based on clinical symptom


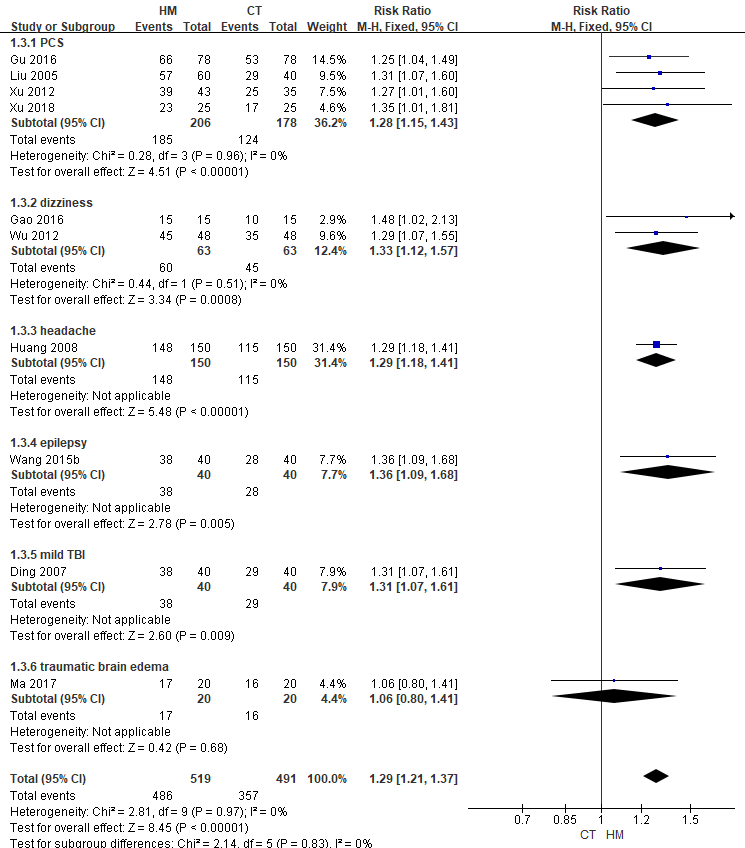


1. Adverse events


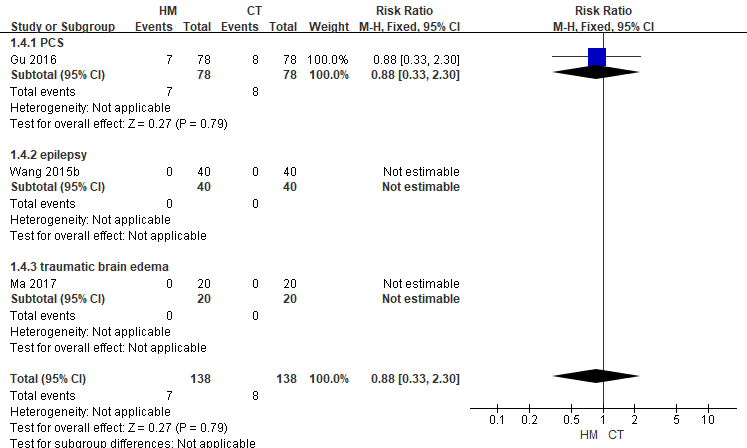


2. Comparison of herbal medicine *versus* placebo

1. Fugi-Meyer assessment


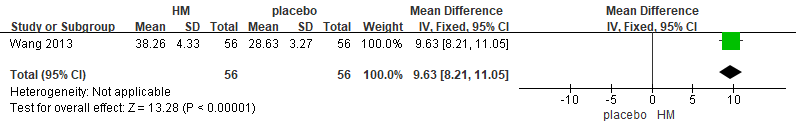


1. Modified Barthel index


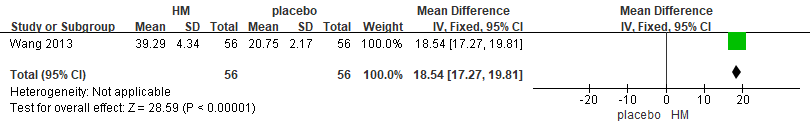


1. Glasgow outcome scale


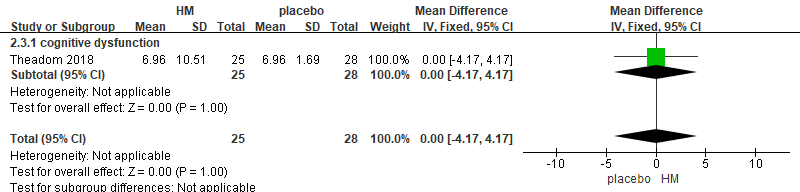


1. Quality of life


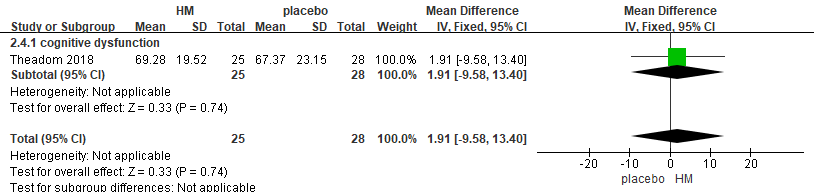


1. Adverse events


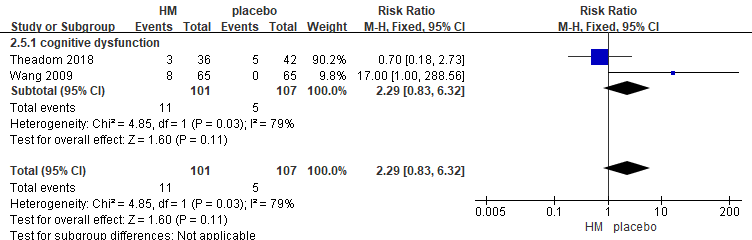


3. Comparison of herbal medicine combined with conventional treatment *versus* conventional treatment alone

1. Activities of daily living


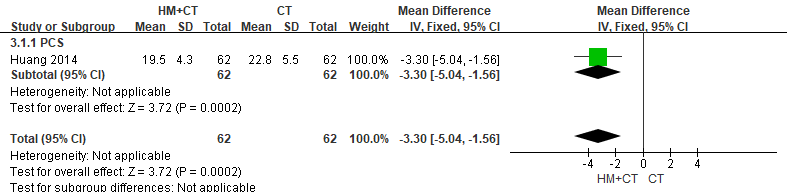


1. Barthel index


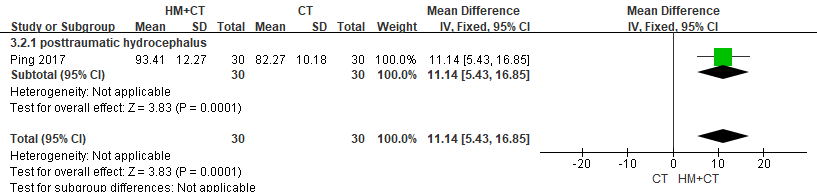


1. SF-36


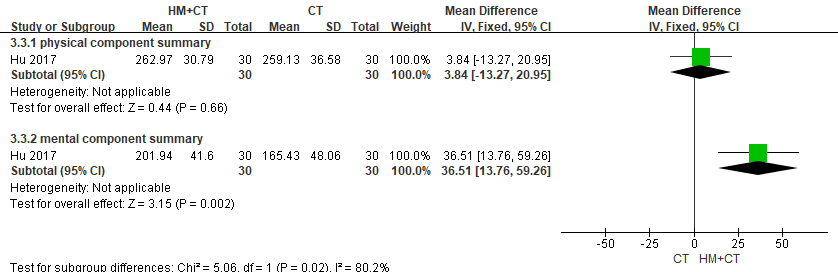


1. GQOLI-74


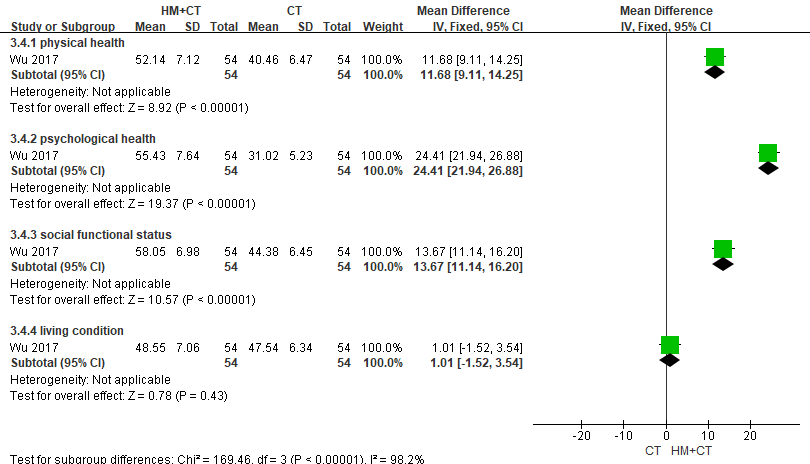


(e) Total effective rate based on clinical symptom


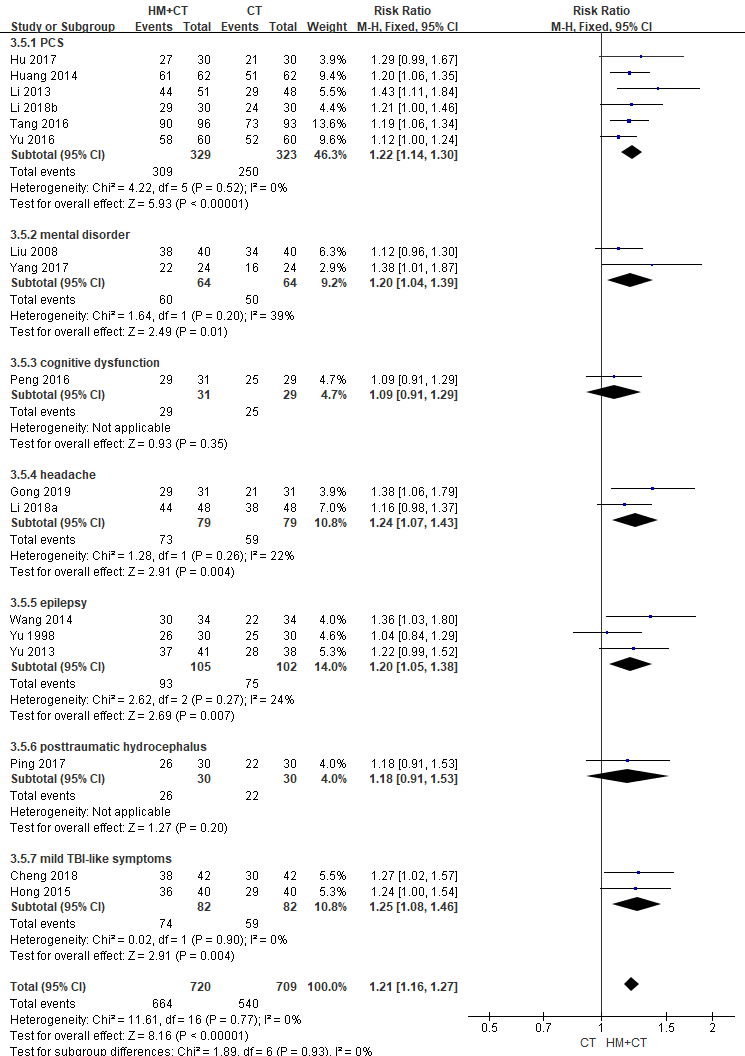


1. Adverse events


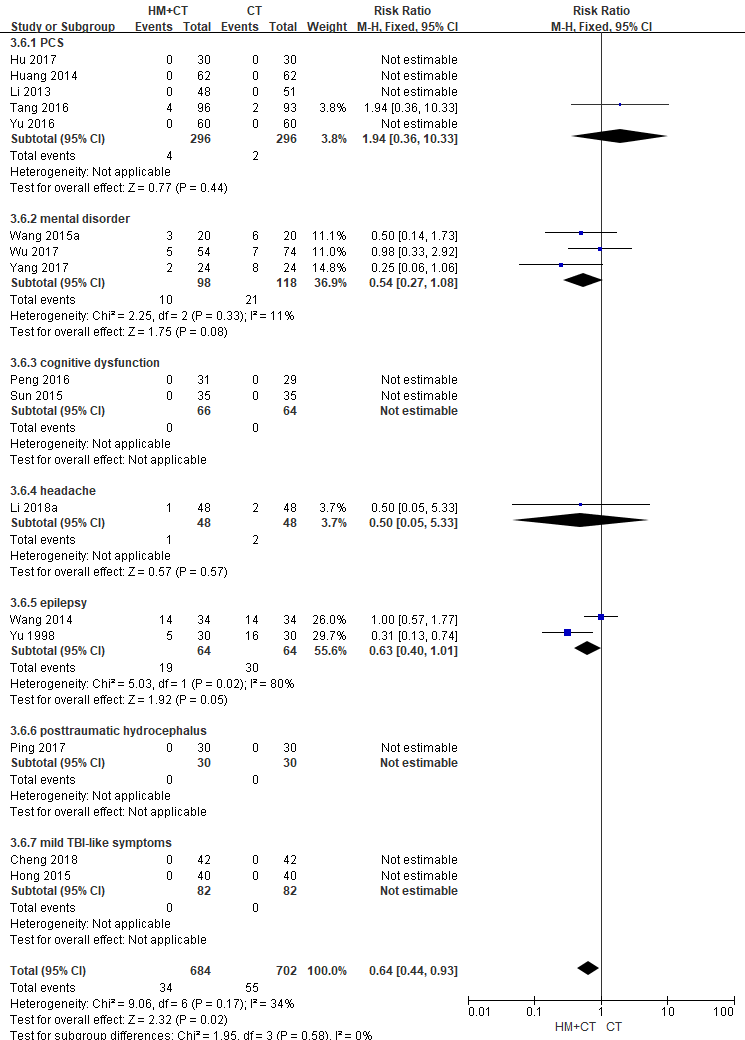


1. Treatment emergent symptom scale


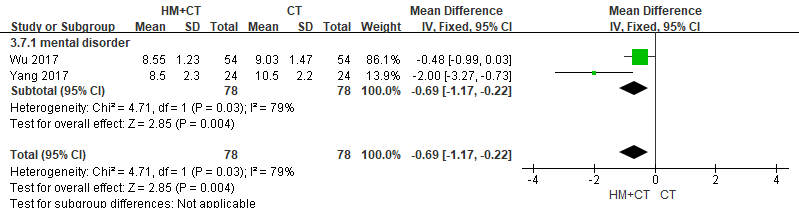


Supplemental Digital Content 4. Results of sensitivity analysis after removal of studies with a high risk of bias

| **Outcomes** |  | **RCT** | **Sample size** | **RR or MD** | **95% CI** | ***I^2^* value** | **Z value** | **P value** |
| --- | --- | --- | --- | --- | --- | --- | --- | --- |
| **HM *versus* placebo** | | | | | | | | |
| GOS | Total (cognitive dysfunction) | 1 | 53 | MD 0.00 | -4.17, 4.17 | NA | 0.00 | 1.00 |
| QoL | Total (cognitive dysfunction) | 1 | 53 | MD 1.91 | -9.58, 13.40 | NA | 0.33 | 0.74 |
| AE | Total (cognitive dysfunction) | 1 | 53 | RR 0.70 | 0.18, 2.73 | NA | 0.51 | 0.61 |
| **HM combined with CT *versus* CT alone** | | | | | | | | |
| SF-36 (physical component summary) | Total (PCS) | 1 | 60 | MD 3.84 | -13.27, 20.95 | NA | 0.44 | 0.66 |
| SF-36 (mental component summary) | Total (PCS) | 1 | 60 | MD 36.51 | 13.76, 59.26 | NA | 3.15 | 0.002 |
| TER (clinical symptom) | Total (PCS) | 1 | 60 | RR 1.29 | 0.99, 1.67 | NA | 1.87 | 0.06 |
| AE | Total (PCS) | 1 | 60 | Not estimable* | Not estimable* | NA | NA | NA |

*It was impossible to calculate the risk ratio because both treatment and control groups reported no adverse events.

**Abbreviations.** AE, adverse event; CI, confidence interval; CT, conventional treatment; GOS, Glasgow outcome scale; HM, herbal medicine; MD, mean difference; NA, not applicable; PCS, post-concussion syndrome; QoL, quality of life; RCT, randomized controlled trial; RR, risk ratio; SF-36, 36-item short form survey; TER, total effective rate

Supplemental Digital Content 5. PRISMA 2009 checklist

| **Section/topic** | **#** | **Checklist item** | **Reported on page #** |
| --- | --- | --- | --- |
| **TITLE** | | |  |
| Title | 1 | Identify the report as a systematic review, meta-analysis, or both. | 1 |
| **ABSTRACT** | | |  |
| Structured summary | 2 | Provide a structured summary including, as applicable: background; objectives; data sources; study eligibility criteria, participants, and interventions; study appraisal and synthesis methods; results; limitations; conclusions and implications of key findings; systematic review registration number. | 2-3 |
| **INTRODUCTION** | | |  |
| Rationale | 3 | Describe the rationale for the review in the context of what is already known. | 4-5 |
| Objectives | 4 | Provide an explicit statement of questions being addressed with reference to participants, interventions, comparisons, outcomes, and study design (PICOS). | 5 |
| **METHODS** | | |  |
| Protocol and registration | 5 | Indicate if a review protocol exists, if and where it can be accessed (e.g., Web address), and, if available, provide registration information including registration number. | 6 |
| Eligibility criteria | 6 | Specify study characteristics (e.g., PICOS, length of follow-up) and report characteristics (e.g., years considered, language, publication status) used as criteria for eligibility, giving rationale. | 6-7 |
| Information sources | 7 | Describe all information sources (e.g., databases with dates of coverage, contact with study authors to identify additional studies) in the search and date last searched. | 6 |
| Search | 8 | Present full electronic search strategy for at least one database, including any limits used, such that it could be repeated. | 6, S1 |
| Study selection | 9 | State the process for selecting studies (i.e., screening, eligibility, included in systematic review, and, if applicable, included in the meta-analysis). | 7-8 |
| Data collection process | 10 | Describe method of data extraction from reports (e.g., piloted forms, independently, in duplicate) and any processes for obtaining and confirming data from investigators. | 8 |
| Data items | 11 | List and define all variables for which data were sought (e.g., PICOS, funding sources) and any assumptions and simplifications made. | 7-8 |
| Risk of bias in individual studies | 12 | Describe methods used for assessing risk of bias of individual studies (including specification of whether this was done at the study or outcome level), and how this information is to be used in any data synthesis. | 8-9 |
| Summary measures | 13 | State the principal summary measures (e.g., risk ratio, difference in means). | 9 |
| Synthesis of results | 14 | Describe the methods of handling data and combining results of studies, if done, including measures of consistency (e.g., I^2^) for each meta-analysis. | 9 |
| Risk of bias across studies | 15 | Specify any assessment of risk of bias that may affect the cumulative evidence (e.g., publication bias, selective reporting within studies). | 8-9 |
| Additional analyses | 16 | Describe methods of additional analyses (e.g., sensitivity or subgroup analyses, meta-regression), if done, indicating which were pre-specified. | 8-9 |
| **RESULTS** | | |  |
| Study selection | 17 | Give numbers of studies screened, assessed for eligibility, and included in the review, with reasons for exclusions at each stage, ideally with a flow diagram. | 10, Figure 1 |
| Study characteristics | 18 | For each study, present characteristics for which data were extracted (e.g., study size, PICOS, follow-up period) and provide the citations. | 10-11,  Table 1, S2,S3 |
| Risk of bias within studies | 19 | Present data on risk of bias of each study and, if available, any outcome level assessment (see item 12). | 11,  Figure 2, 3 |
| Results of individual studies | 20 | For all outcomes considered (benefits or harms), present, for each study: (a) simple summary data for each intervention group (b) effect estimates and confidence intervals, ideally with a forest plot. | 11-14  Table 1,S2,S3 |
| Synthesis of results | 21 | Present results of each meta-analysis done, including confidence intervals and measures of consistency. | 11-14,  Table 2,S3 |
| Risk of bias across studies | 22 | Present results of any assessment of risk of bias across studies (see Item 15). | 14  Figure 2,3 |
| Additional analysis | 23 | Give results of additional analyses, if done (e.g., sensitivity or subgroup analyses, meta-regression [see Item 16]). | 11-12  S4 |
| **DISCUSSION** | | |  |
| Summary of evidence | 24 | Summarize the main findings including the strength of evidence for each main outcome; consider their relevance to key groups (e.g., healthcare providers, users, and policy makers). | 16  Table 2 |
| Limitations | 25 | Discuss limitations at study and outcome level (e.g., risk of bias), and at review-level (e.g., incomplete retrieval of identified research, reporting bias). | 17 |
| Conclusions | 26 | Provide a general interpretation of the results in the context of other evidence, and implications for future research. | 17-18 |
| **FUNDING** | | |  |
| Funding | 27 | Describe sources of funding for the systematic review and other support (e.g., supply of data); role of funders for the systematic review. | 24 |
